# Supplementary material for: Differential whole-genome doubling and homologous recombination deficiencies across breast cancer subtypes from the Taiwanese population
Source: Commun Biol. 2021 Sep 9;4:1052. doi: 10.1038/s42003-021-02597-x (PMC8429690; doi:10.1038/s42003-021-02597-x)
Supplement: Supplementary file 2 — Supplementary Information [file 42003_2021_2597_MOESM2_ESM.pdf]

Supplementary Information (Figures and Notes)

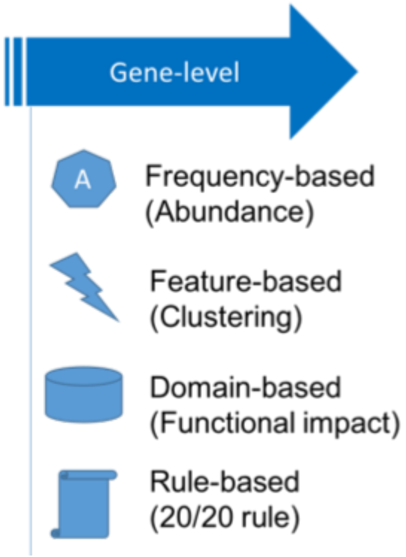

Supplementary Figure 1. Schematic representation of the significantly mutated gene (SMG) analytic strategy. Overall schema showing how we used different algorithms to identify our SMG consensus list and the driver mutations. Gene-level approaches were employed as indicated (discussion of the SMGs can be found in Supplementary Notes). Software modules in the gene-level approach included: dNdScv (frequency-based approach), OncodriveCLUST (feature-based approach), OncodriveFML (domain-based approach), and 20/20+ (rule-based approach).

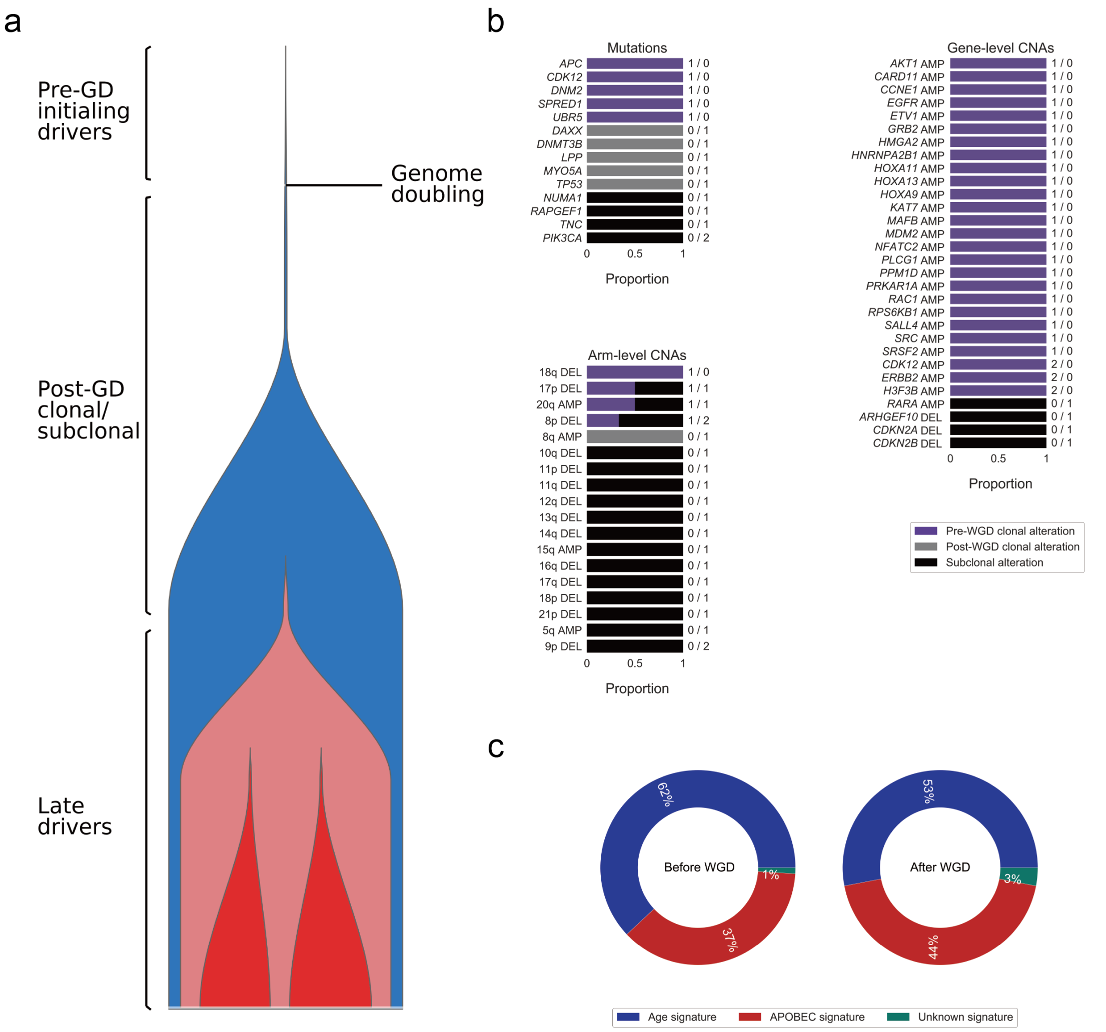

Supplementary Figure 2. Timing of somatic events in human epidermal growth factor receptor-positive breast cancer (HER2+; N=3). a A diagram of tumor evolution in the HER2+ subtype shows the approximate timing of genomic alterations with respect to the cancer's lifetime. **b** The timing of mutations and copy number events is shown as bars indicating whether the events are clonal or subclonal. Clonal mutations and chromosome arm events are further designated as early or late with respect to whole genome doubling (WGD). The number of samples harboring mutations and copy

number alterations (CNAs; pre-GD and post-GD) is indicated on the right side of the bars. **c** Pie charts show the percentage of estimated mutations for each of three signatures, averaged across the HER2+ cohort. Only genes that were mutated in the Cancer Gene Census or canonical signaling pathways in the cohort are shown. AMP: amplification; DEL: deletion.

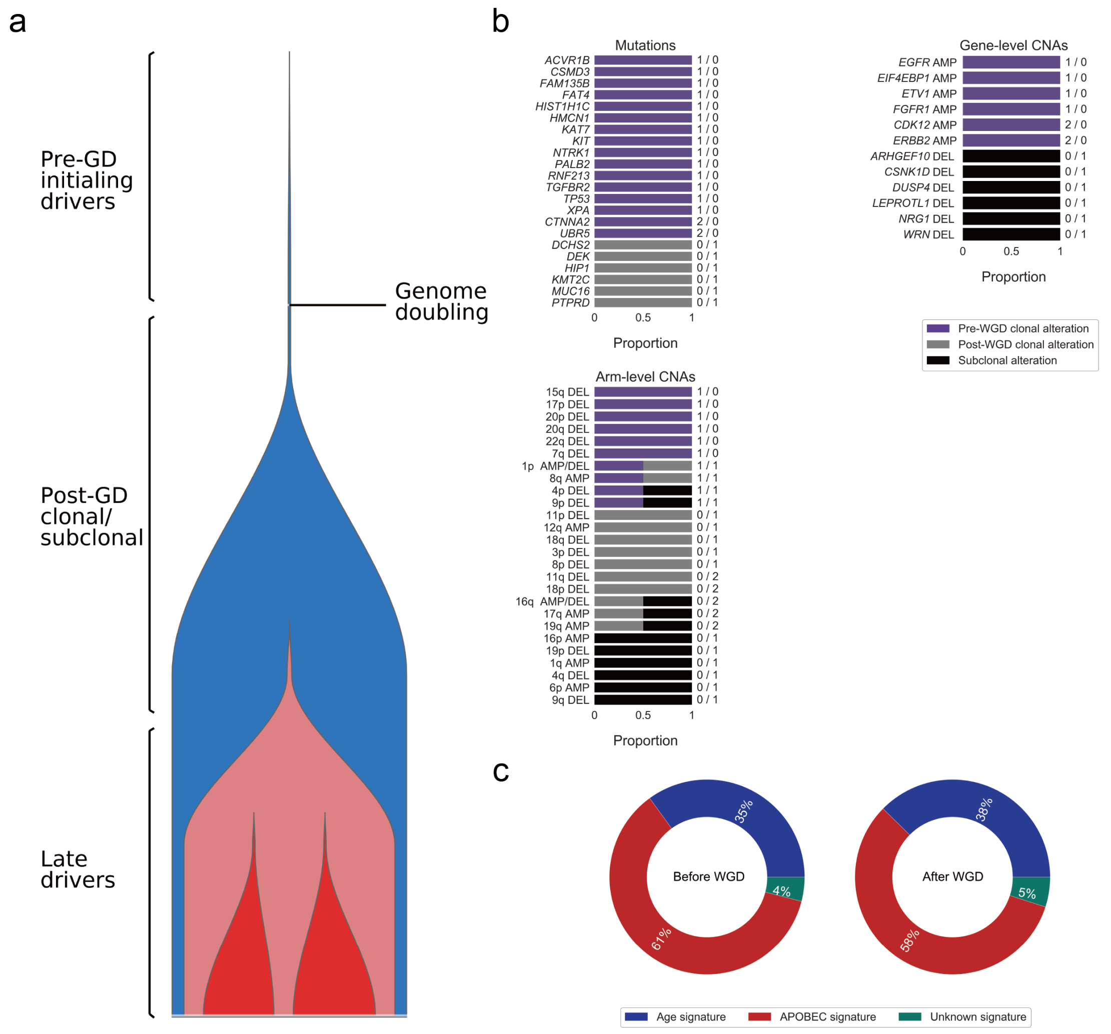

Supplementary Figure 3. Timing of somatic events in hormone receptor-positive/human epidermal growth factor receptor-positive breast cancer (HR+/HER2+; N=3). a A diagram of tumor evolution in the HR+/HER2+ subtype shows the approximate timing of genomic alterations with respect to the cancer's lifetime. **b** The timing of mutations and copy number events is shown as bars indicating whether the events are clonal or subclonal. Clonal mutations and chromosome arm events are further designated as early or late with respect to whole genome doubling

(WGD). The number of samples harboring mutations and copy number alterations (CNAs; pre-GD and post-GD) is indicated on the right side of the bars. **c** Pie charts show the percentage of estimated mutations for each of three signatures, averaged across the HR+/HER2+ cohort. Only genes that were mutated in the Cancer Gene Census or canonical signaling pathways in the cohort are shown. AMP: amplification; DEL: deletion.

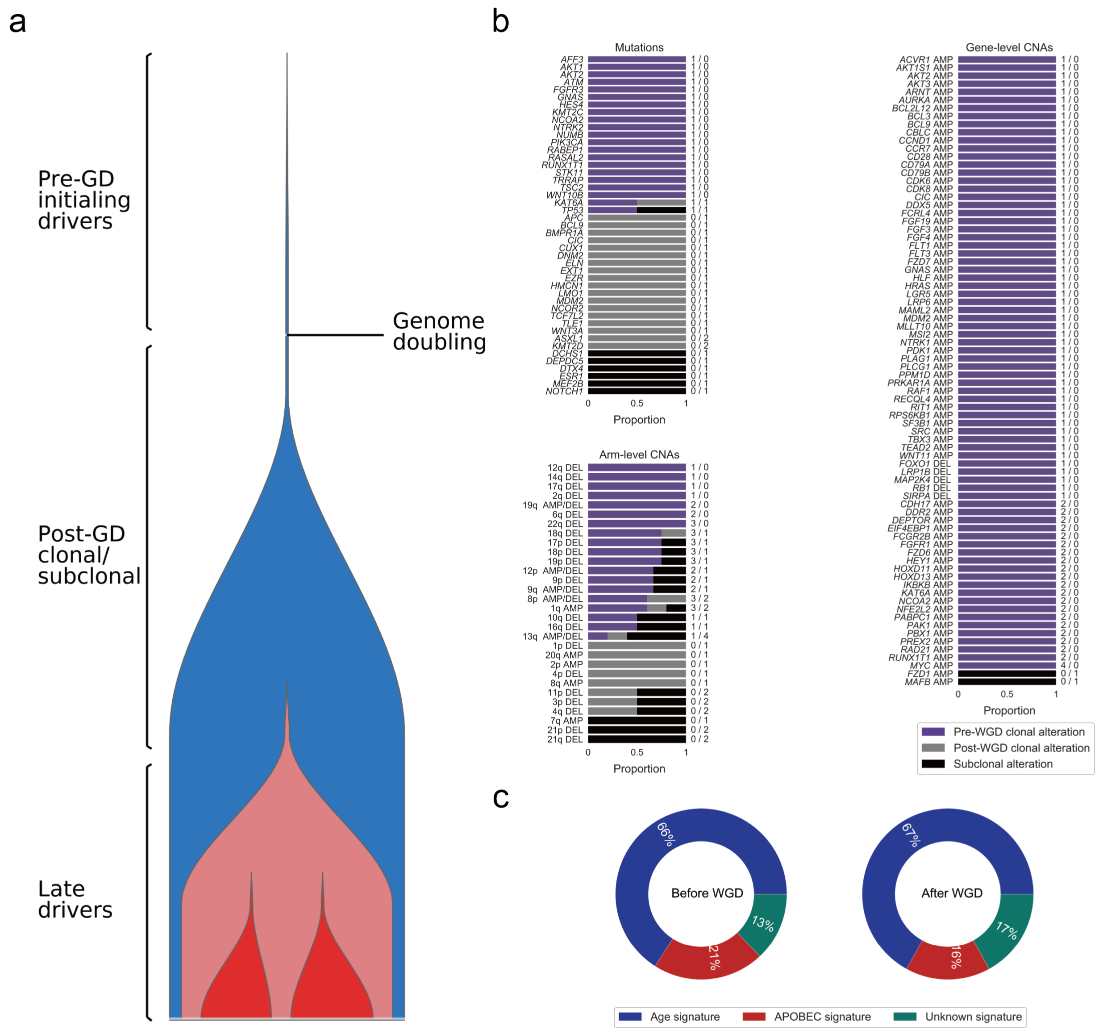

Supplementary Figure 4. Timing of somatic events in hormone receptor-positive/human epidermal growth factor receptor-negative breast cancer (HR+/HER2-; N=9). a A diagram of tumor evolution in the HR+/HER2- subtype shows the approximate timing of genomic alterations with respect to the cancer's lifetime. **b** The timing of mutations and copy number events is shown as bars indicating whether the events are clonal or subclonal. Clonal mutations and chromosome arm events are further designated as early or late with respect to whole genome doubling

(WGD). The number of samples harboring mutations and copy number alterations (CNAs; pre-GD and post-GD) is indicated on the right side of the bars. **c** Pie charts show the percentage of estimated mutations for each signature, averaged across the HR+/HER2- cohort. Only genes that were mutated in the Cancer Gene Census or canonical signaling pathways in the cohort are shown. AMP: amplification; DEL: deletion.

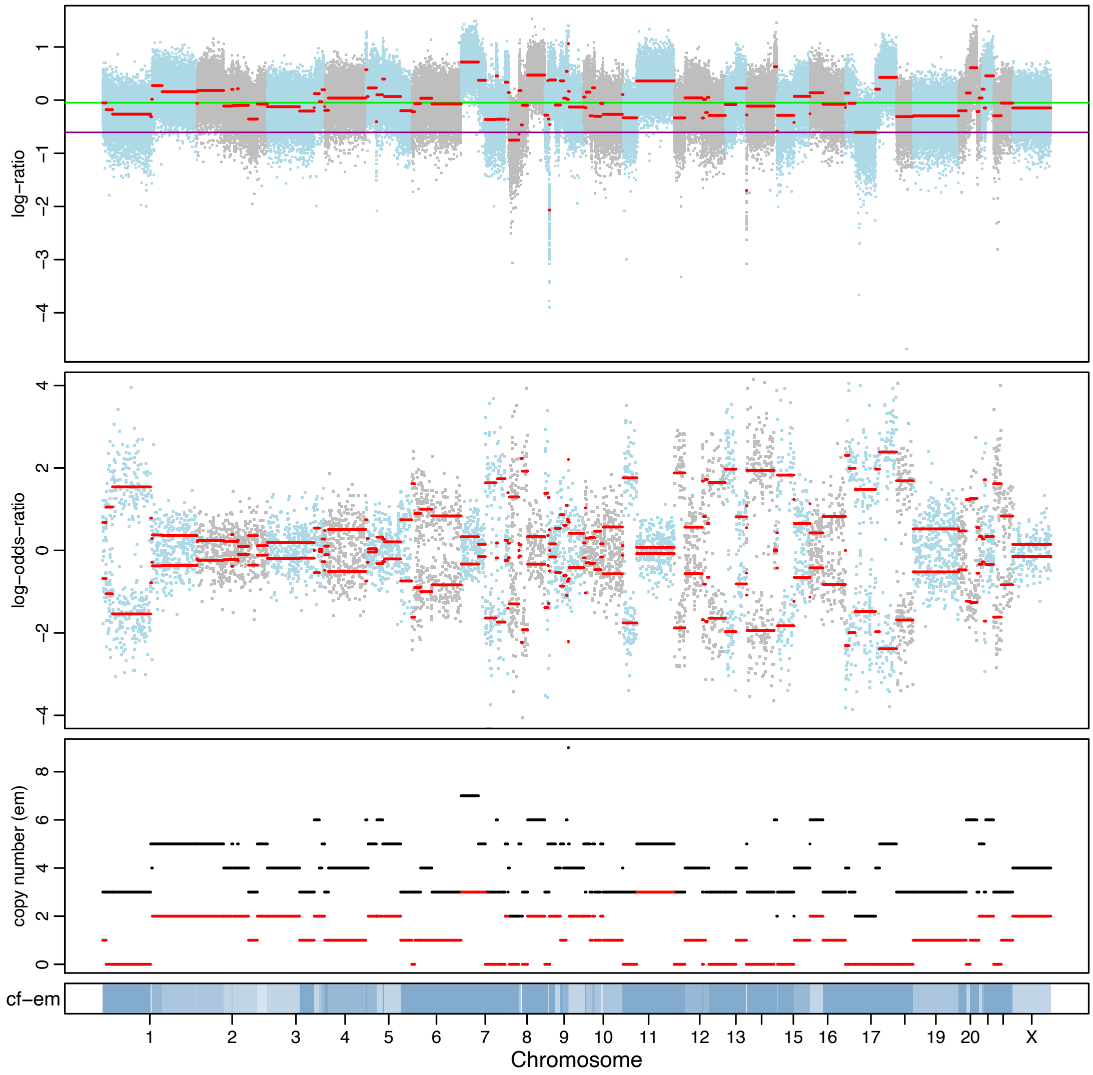

Supplementary Figure 5. Integrated visualization of FACETS analysis of WGD sample (CBCP00004). With chromosomes alternating in blue and gray, the top plot indicates copy number log-ratio (logR), and the second plot indicates allele-specific log-odds-ratio data (logOR). The third plot indicates integer (total and minor) copy number estimation (em). The bottom plot displays the cellular fraction estimation (cf-em), indicating both clonal and subclonal copy number events.

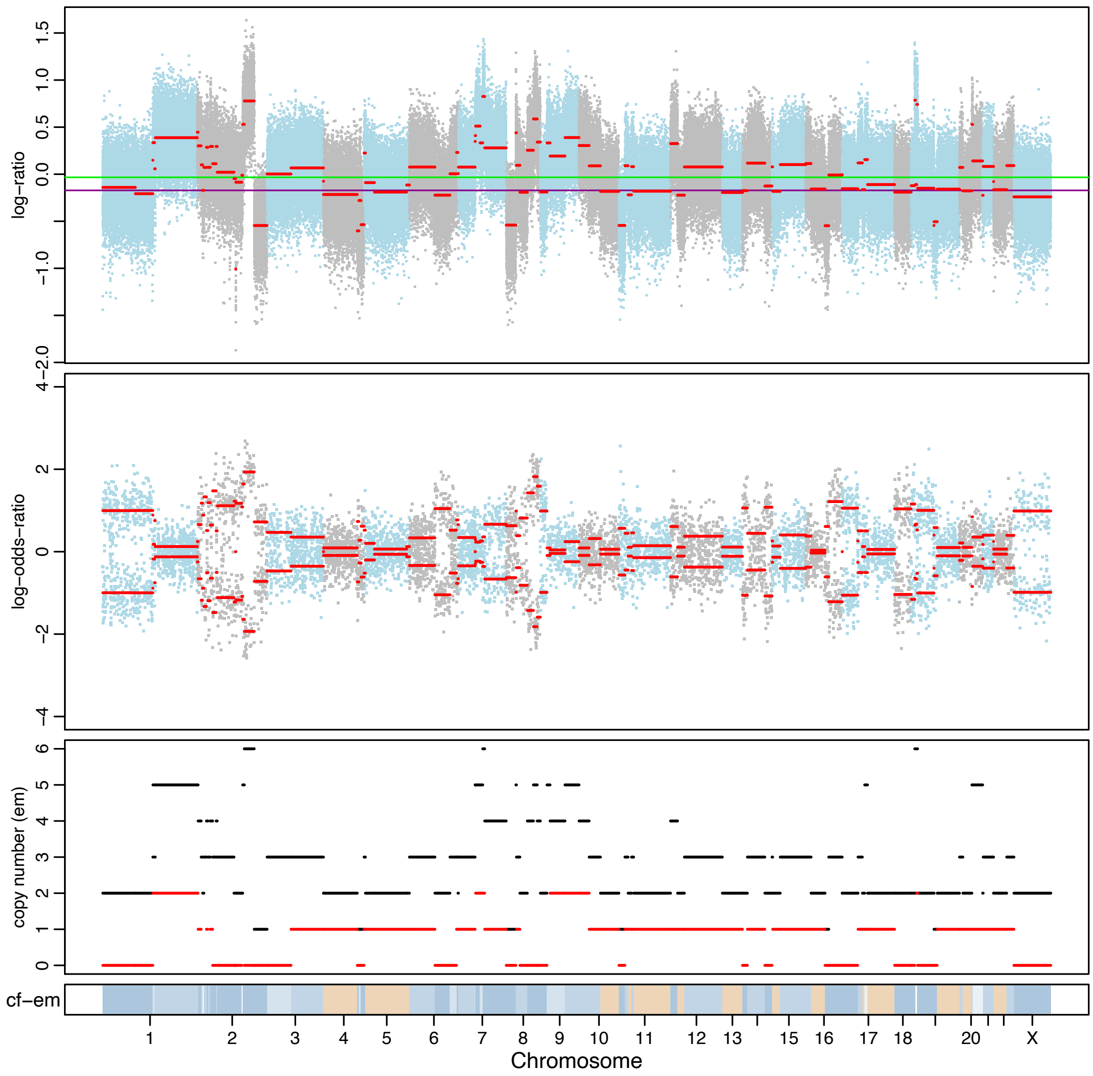

Supplementary Figure 6. Integrated visualization of FACETS analysis of WGD sample (CBCP00005). With chromosomes alternating in blue and gray, the top plot indicates copy number log-ratio (logR), and the second plot indicates allele-specific log-odds-ratio data (logOR). The third plot indicates integer (total and minor) copy number estimation (em). The bottom plot displays the cellular fraction estimation (cf-em), indicating both clonal and subclonal copy number events.

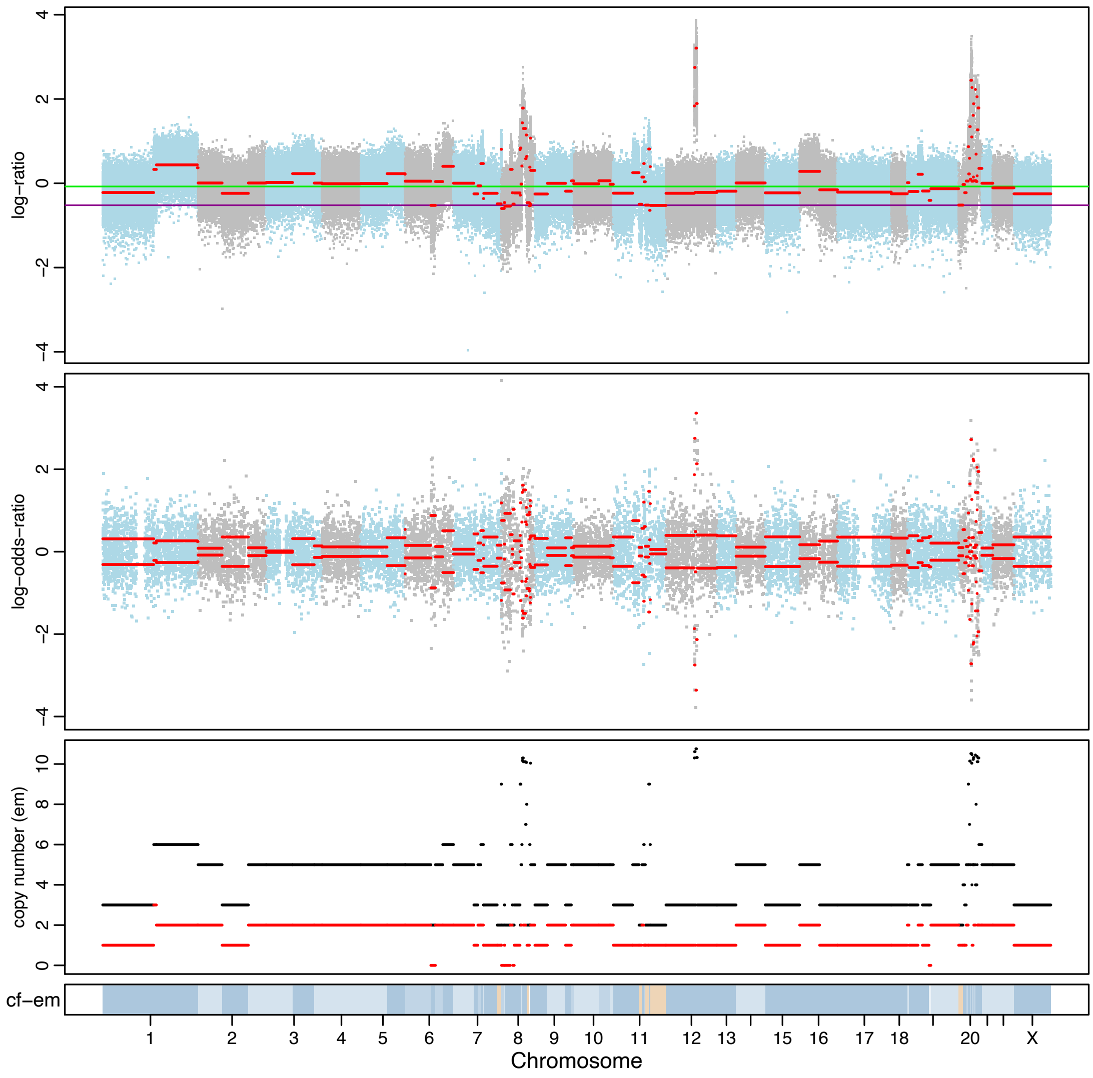

Supplementary Figure 7. Integrated visualization of FACETS analysis of WGD sample (CBCP00007). With chromosomes alternating in blue and gray, the top plot indicates copy number log-ratio (logR), and the second plot indicates allele-specific log-odds-ratio data (logOR). The third plot indicates integer (total and minor) copy number estimation (em). The bottom plot displays the cellular fraction estimation (cf-em), indicating both clonal and subclonal copy number events.

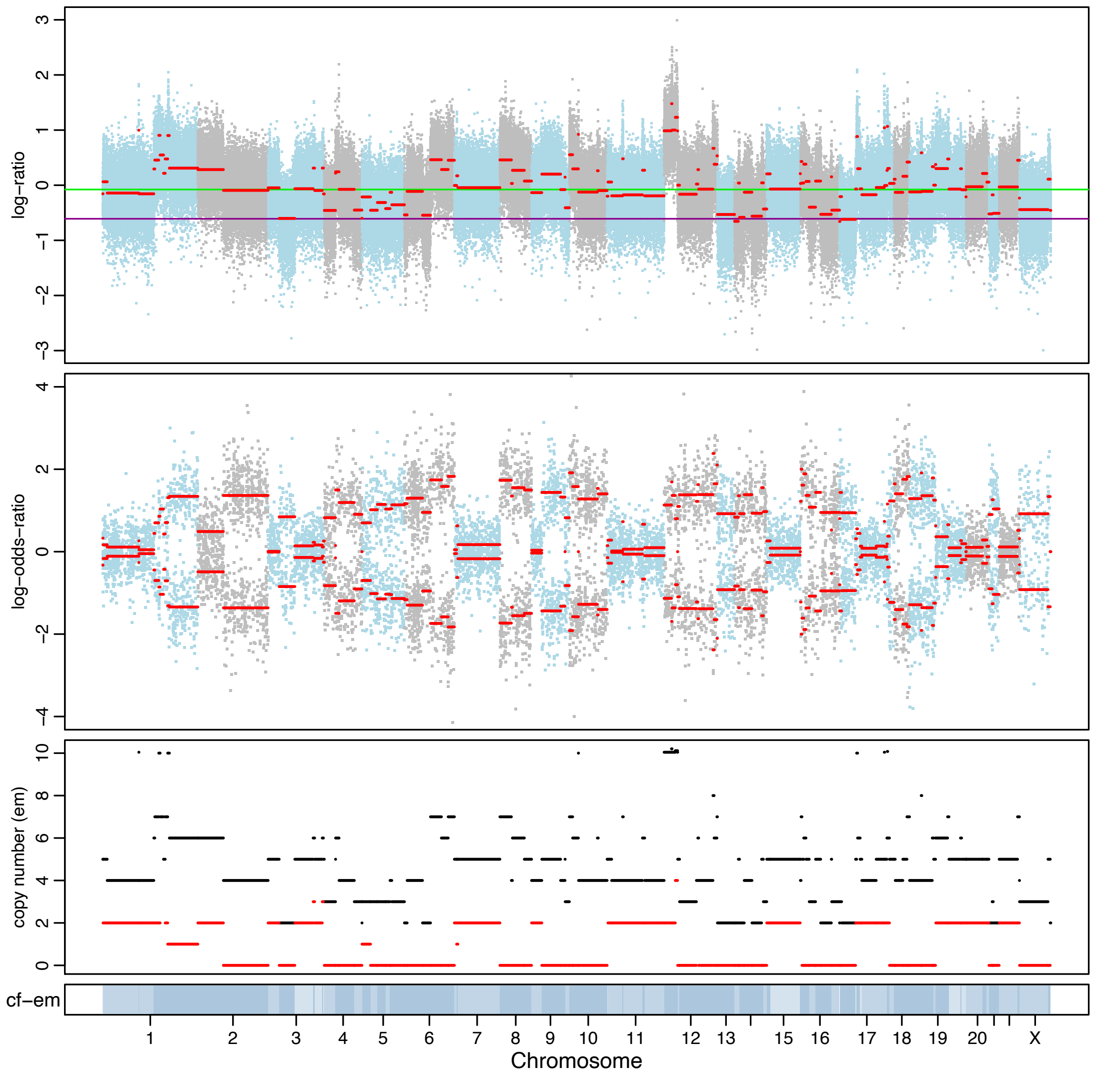

Supplementary Figure 8. Integrated visualization of FACETS analysis of WGD sample (CBCP00014). With chromosomes alternating in blue and gray, the top plot indicates copy number log-ratio (logR), and the second plot indicates allele-specific log-odds-ratio data (logOR). The third plot indicates integer (total and minor) copy number estimation (em). The bottom plot displays the cellular fraction estimation (cf-em), indicating both clonal and subclonal copy number events.

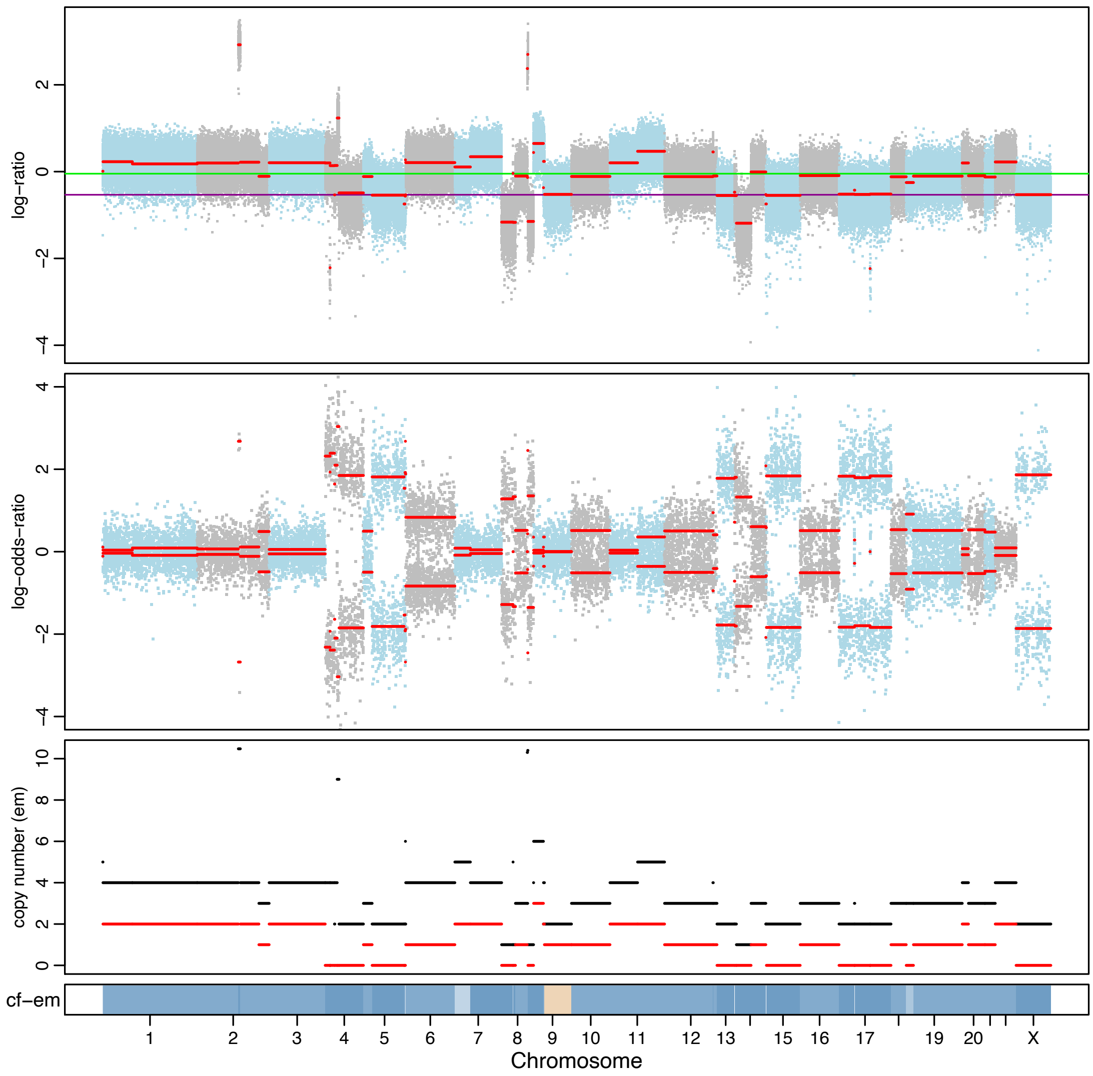

Supplementary Figure 9. Integrated visualization of FACETS analysis of WGD sample (CBCP00022). With chromosomes alternating in blue and gray, the top plot indicates copy number log-ratio (logR), and the second plot indicates allele-specific log-odds-ratio data (logOR). The third plot indicates integer (total and minor) copy number estimation (em). The bottom plot displays the cellular fraction estimation (cf-em), indicating both clonal and subclonal copy number events.

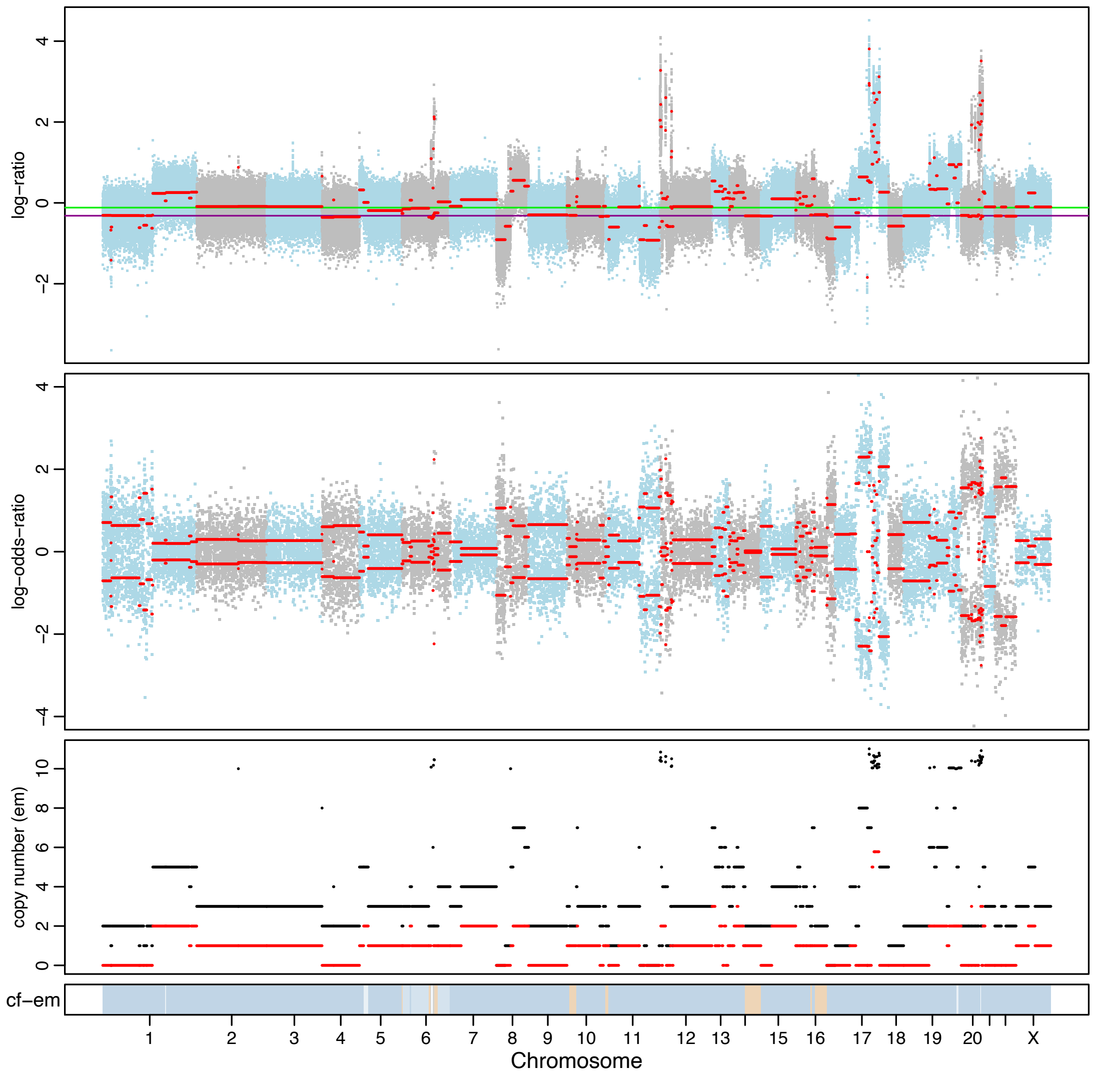

Supplementary Figure 10. Integrated visualization of FACETS analysis of WGD sample (CBCP00027). With chromosomes alternating in blue and gray, the top plot indicates copy number log-ratio (logR), and the second plot indicates allele-specific log-odds-ratio data (logOR). The third plot indicates integer (total and minor) copy number estimation (em). The bottom plot displays the cellular fraction estimation (cf-em), indicating both clonal and subclonal copy number events.

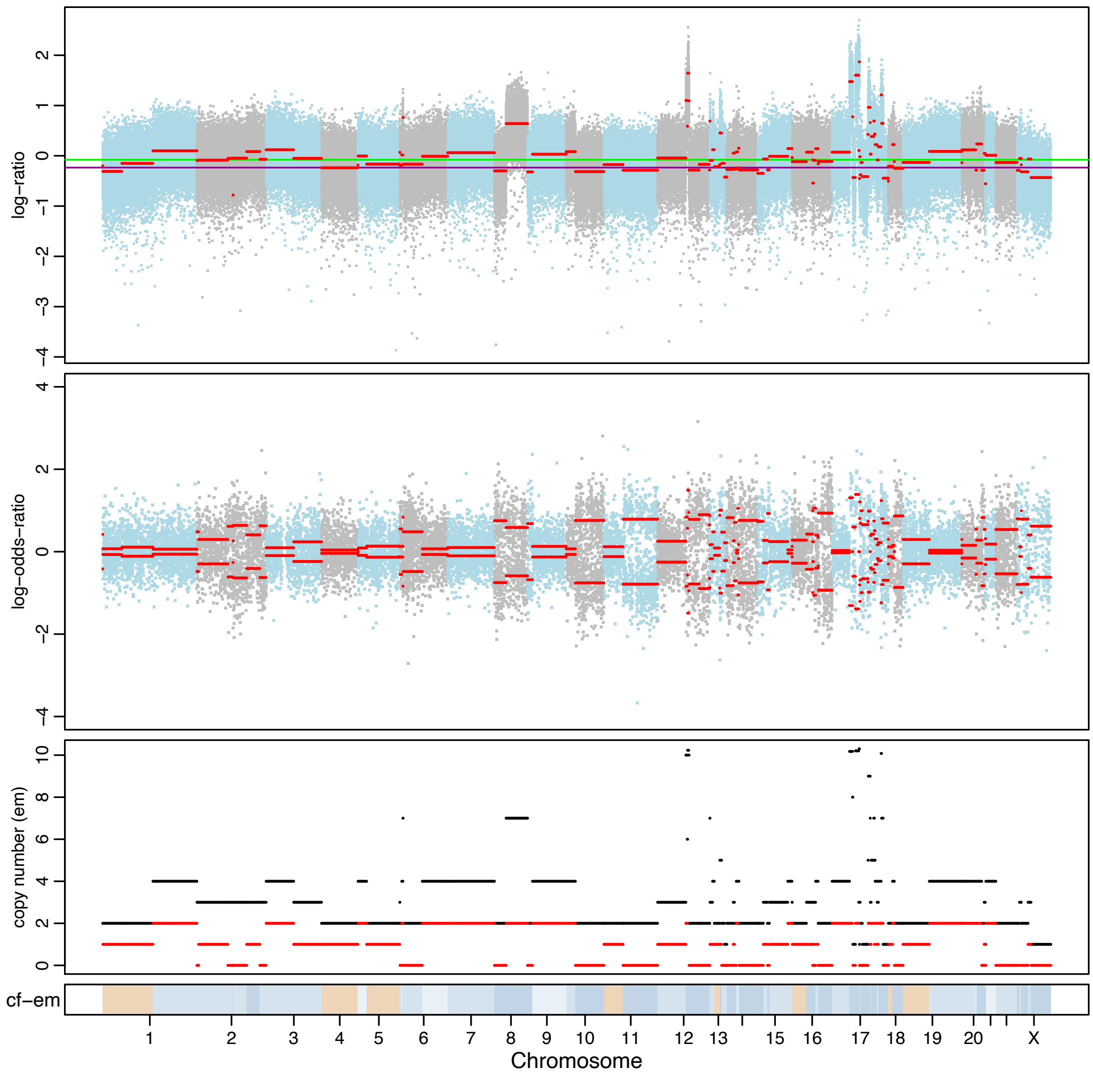

Supplementary Figure 11. Integrated visualization of FACETS analysis of WGD sample (CBCP00033). With chromosomes alternating in blue and gray, the top plot indicates copy number log-ratio (logR), and the second plot indicates allele-specific log-odds-ratio data (logOR). The third plot indicates integer (total and minor) copy number estimation (em). The bottom plot displays the cellular fraction estimation (cf-em), indicating both clonal and subclonal copy number events.

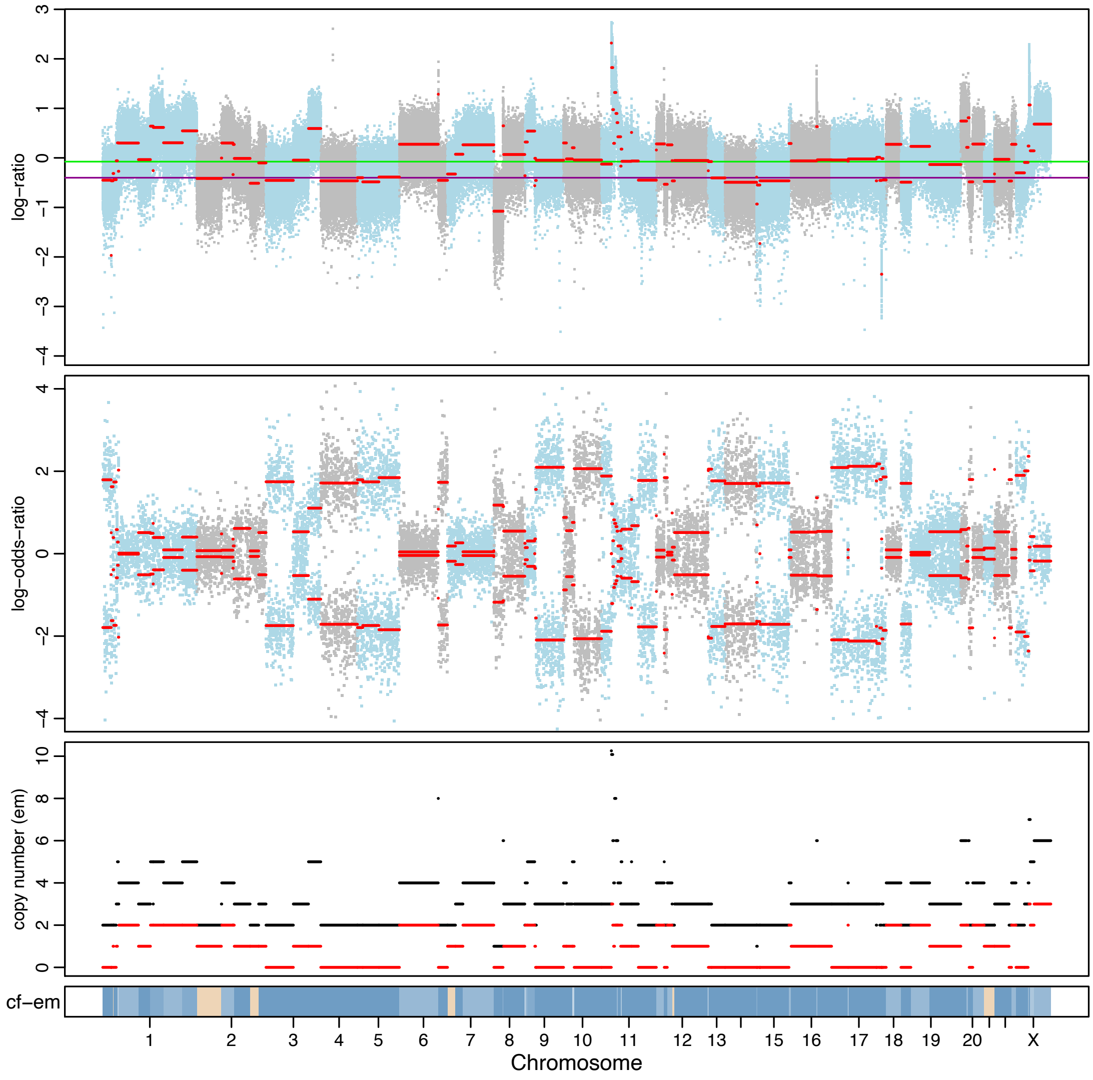

Supplementary Figure 12. Integrated visualization of FACETS analysis of WGD sample (CBCP00035). With chromosomes alternating in blue and gray, the top plot indicates copy number log-ratio (logR), and the second plot indicates allele-specific log-odds-ratio data (logOR). The third plot indicates integer (total and minor) copy number estimation (em). The bottom plot displays the cellular fraction estimation (cf-em), indicating both clonal and subclonal copy number events.

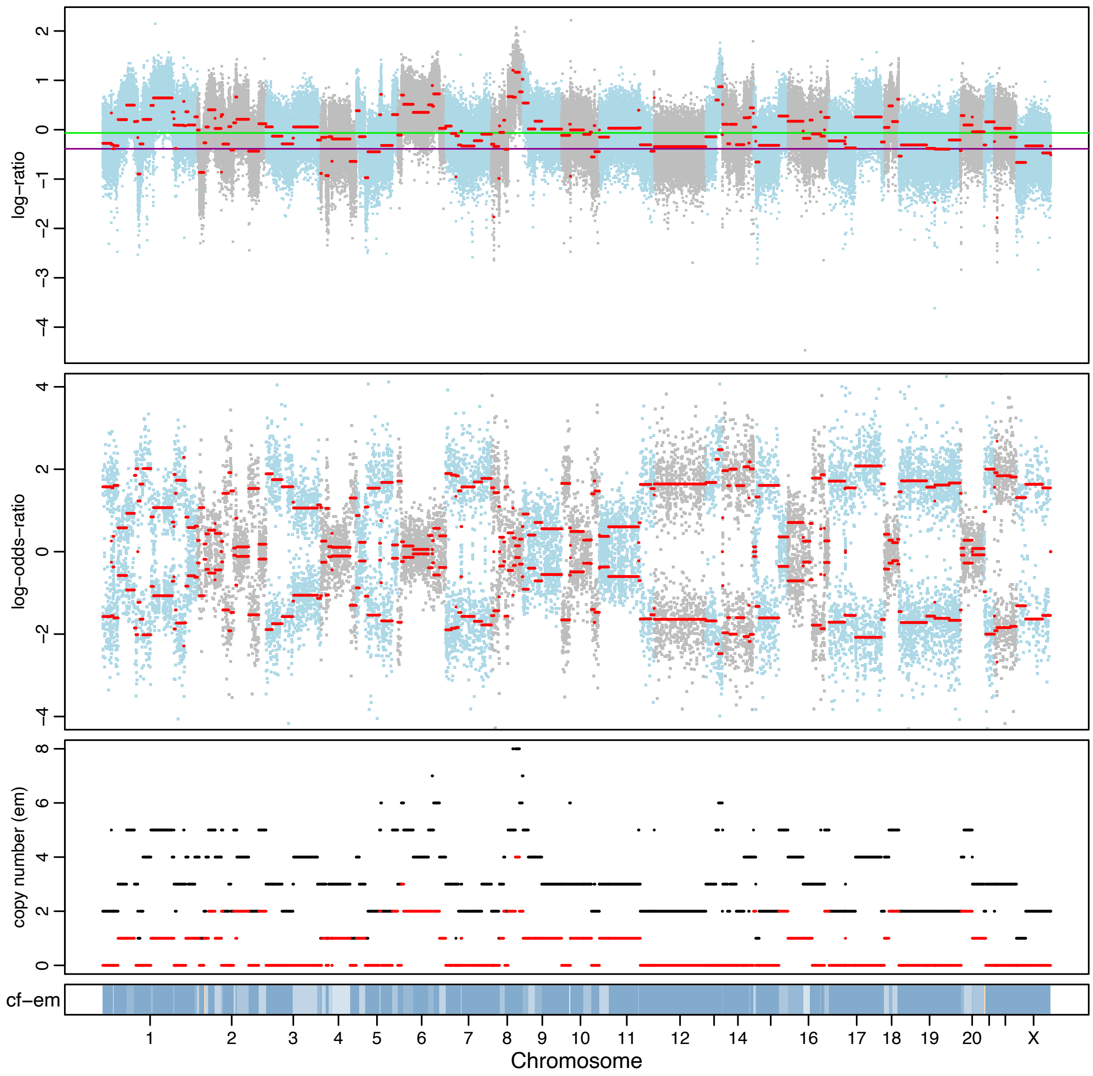

Supplementary Figure 13. Integrated visualization of FACETS analysis of WGD sample (CBCP00037). With chromosomes alternating in blue and gray, the top plot indicates copy number log-ratio (logR), and the second plot indicates allele-specific log-odds-ratio data (logOR). The third plot indicates integer (total and minor) copy number estimation (em). The bottom plot displays the cellular fraction estimation (cf-em), indicating both clonal and subclonal copy number events.

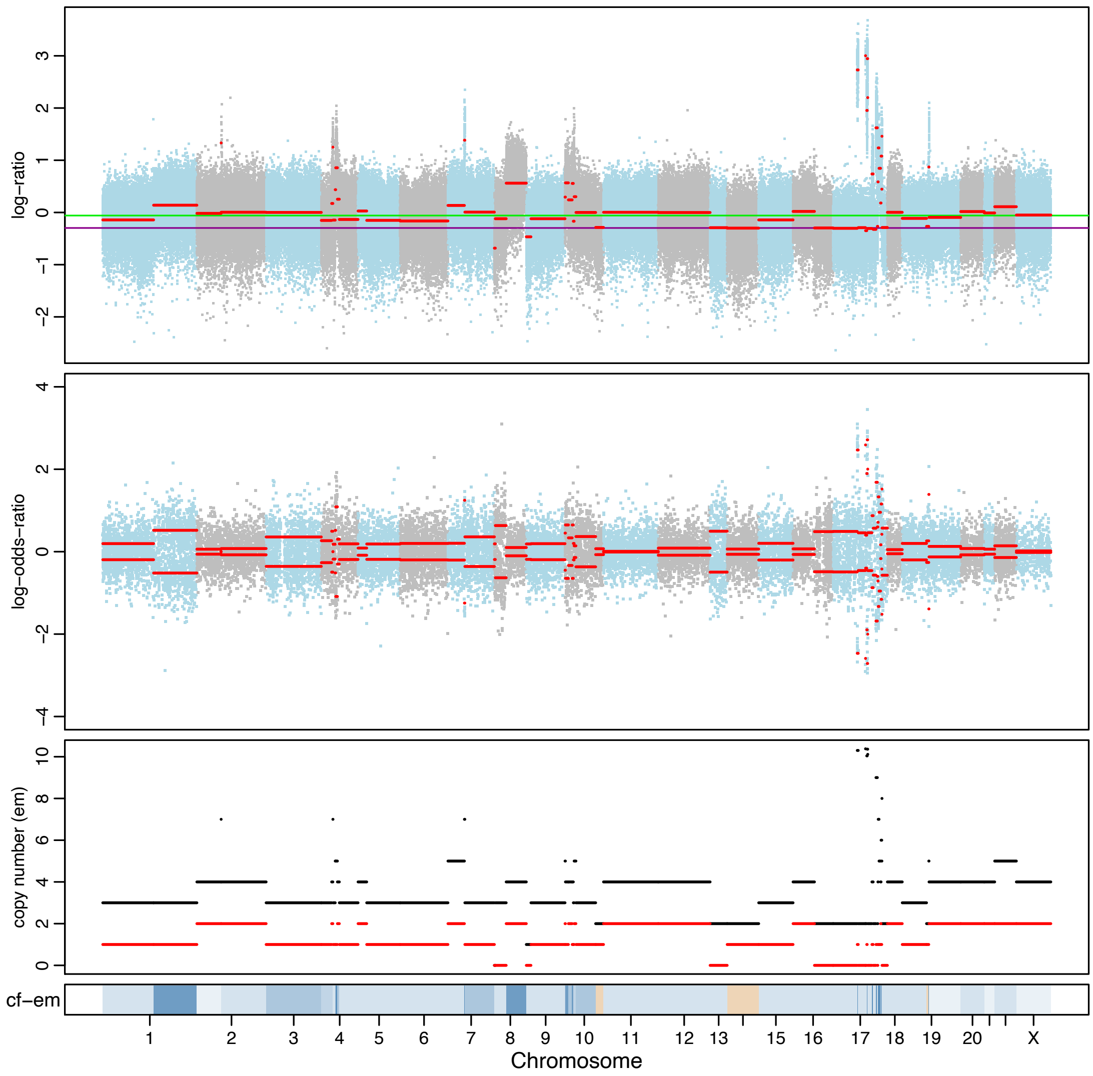

Supplementary Figure 14. Integrated visualization of FACETS analysis of WGD sample (CBCP00042). With chromosomes alternating in blue and gray, the top plot indicates copy number log-ratio (logR), and the second plot indicates allele-specific log-odds-ratio data (logOR). The third plot indicates integer (total and minor) copy number estimation (em). The bottom plot displays the cellular fraction estimation (cf-em), indicating both clonal and subclonal copy number events.

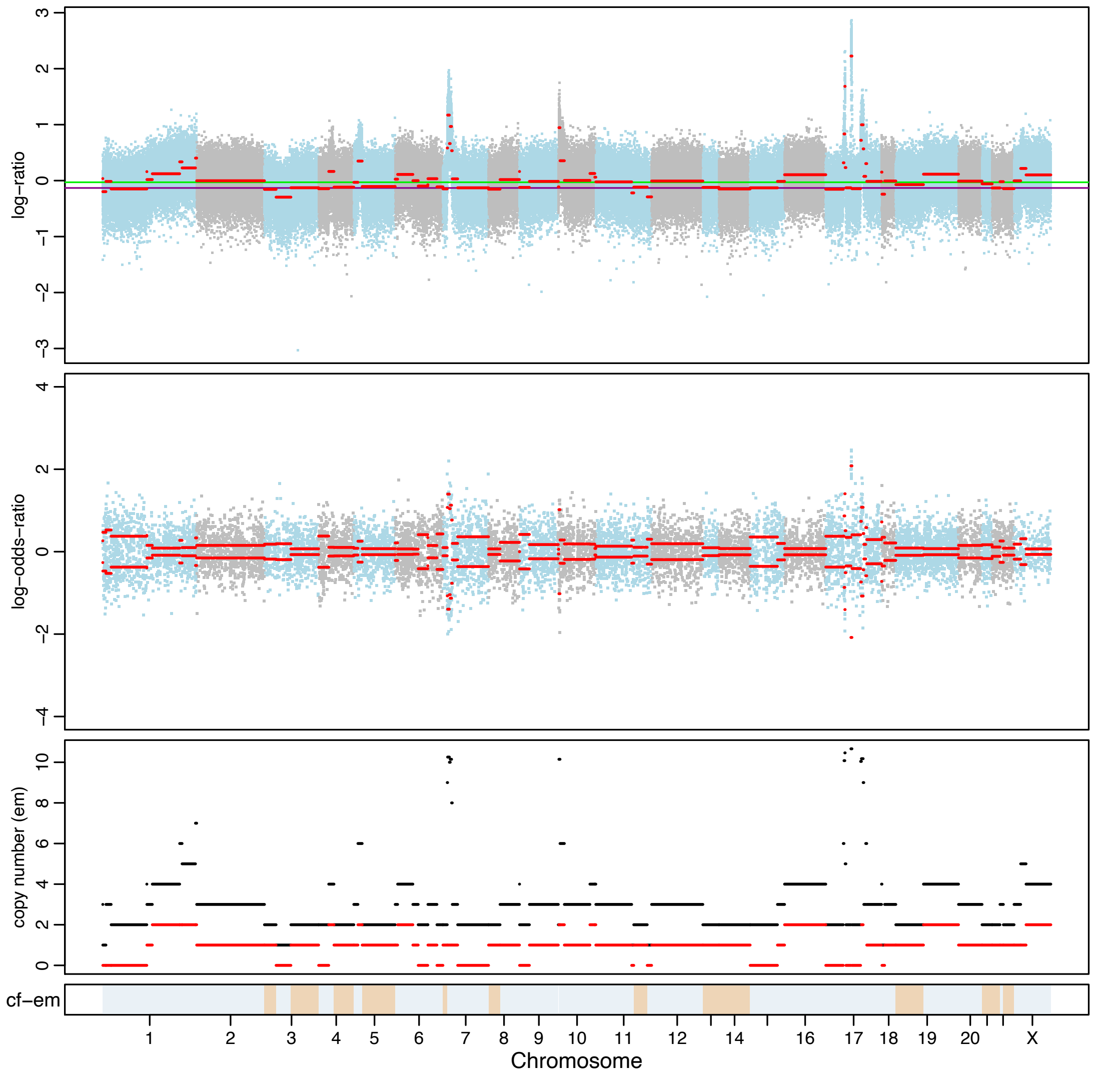

Supplementary Figure 15. Integrated visualization of FACETS analysis of WGD sample (CBCP00049). With chromosomes alternating in blue and gray, the top plot indicates copy number log-ratio (logR), and the second plot indicates allele-specific log-odds-ratio data (logOR). The third plot indicates integer (total and minor) copy number estimation (em). The bottom plot displays the cellular fraction estimation (cf-em), indicating both clonal and subclonal copy number events.

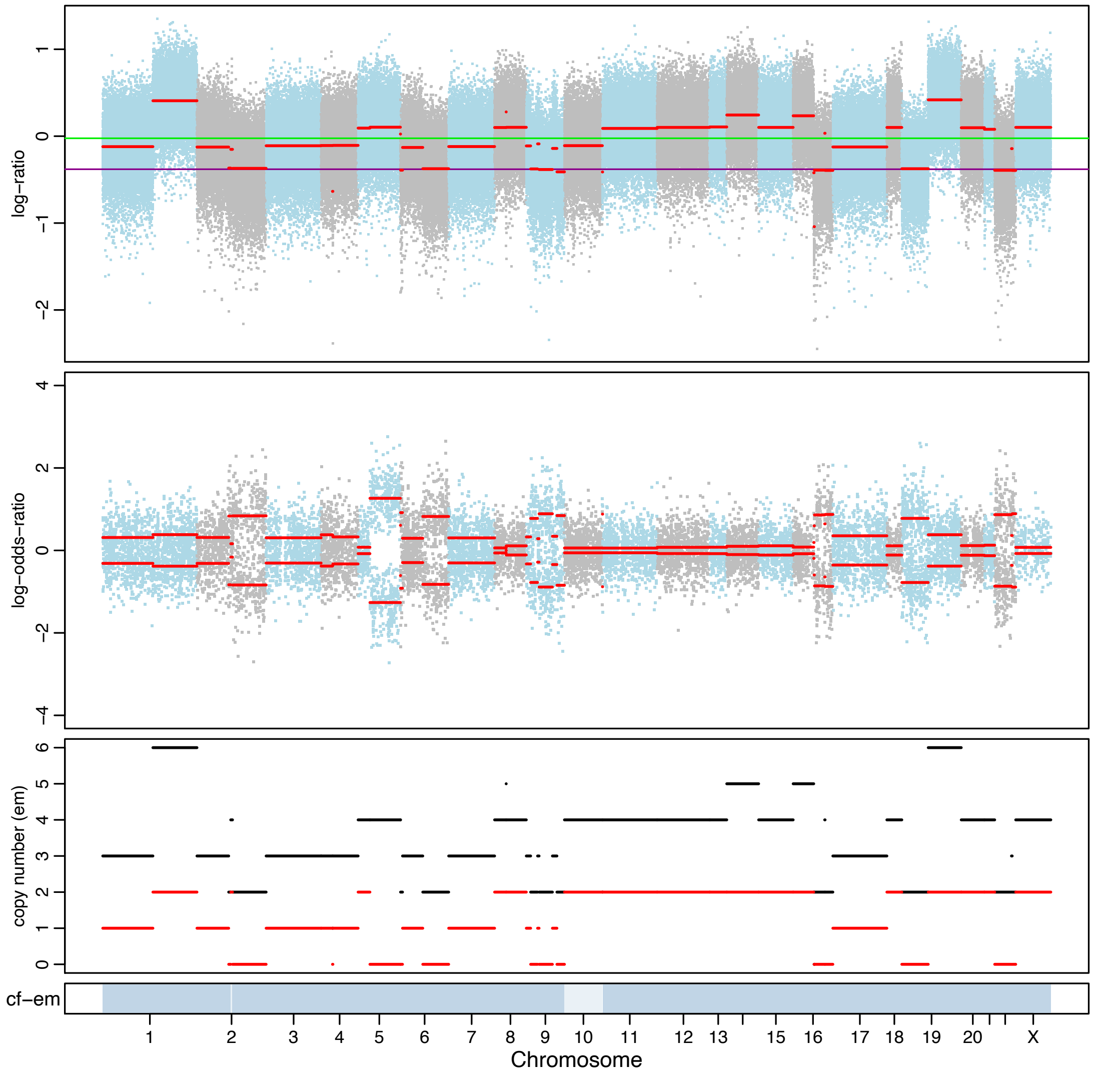

Supplementary Figure 16. Integrated visualization of FACETS analysis of WGD sample (CBCP00050). With chromosomes alternating in blue and gray, the top plot indicates copy number log-ratio (logR), and the second plot indicates allele-specific log-odds-ratio data (logOR). The third plot indicates integer (total and minor) copy number estimation (em). The bottom plot displays the cellular fraction estimation (cf-em), indicating both clonal and subclonal copy number events.

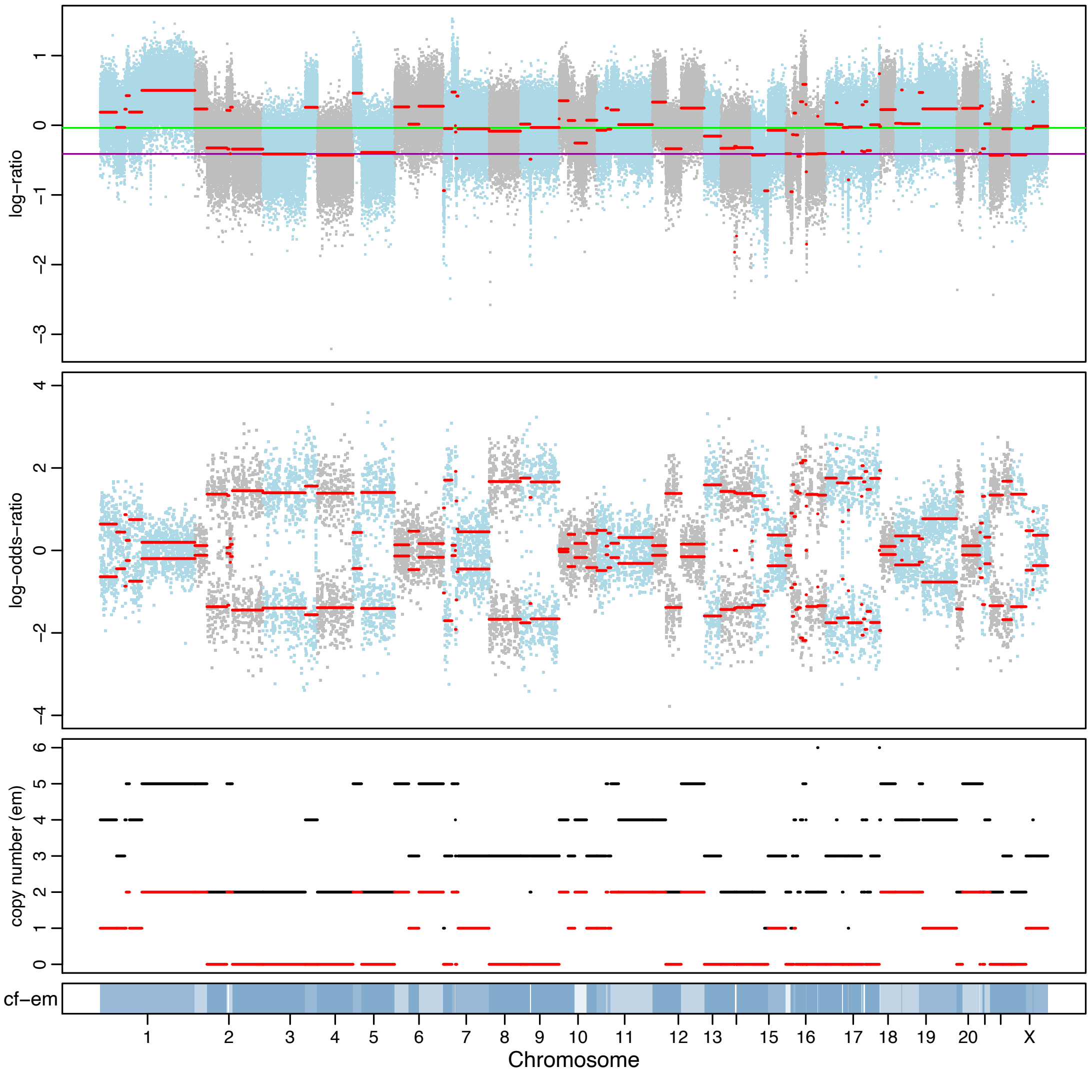

Supplementary Figure 17. Integrated visualization of FACETS analysis of WGD sample (CBCP00053). With chromosomes alternating in blue and gray, the top plot indicates copy number log-ratio (logR), and the second plot indicates allele-specific log-odds-ratio data (logOR). The third plot indicates integer (total and minor) copy number estimation (em). The bottom plot displays the cellular fraction estimation (cf-em), indicating both clonal and subclonal copy number events.

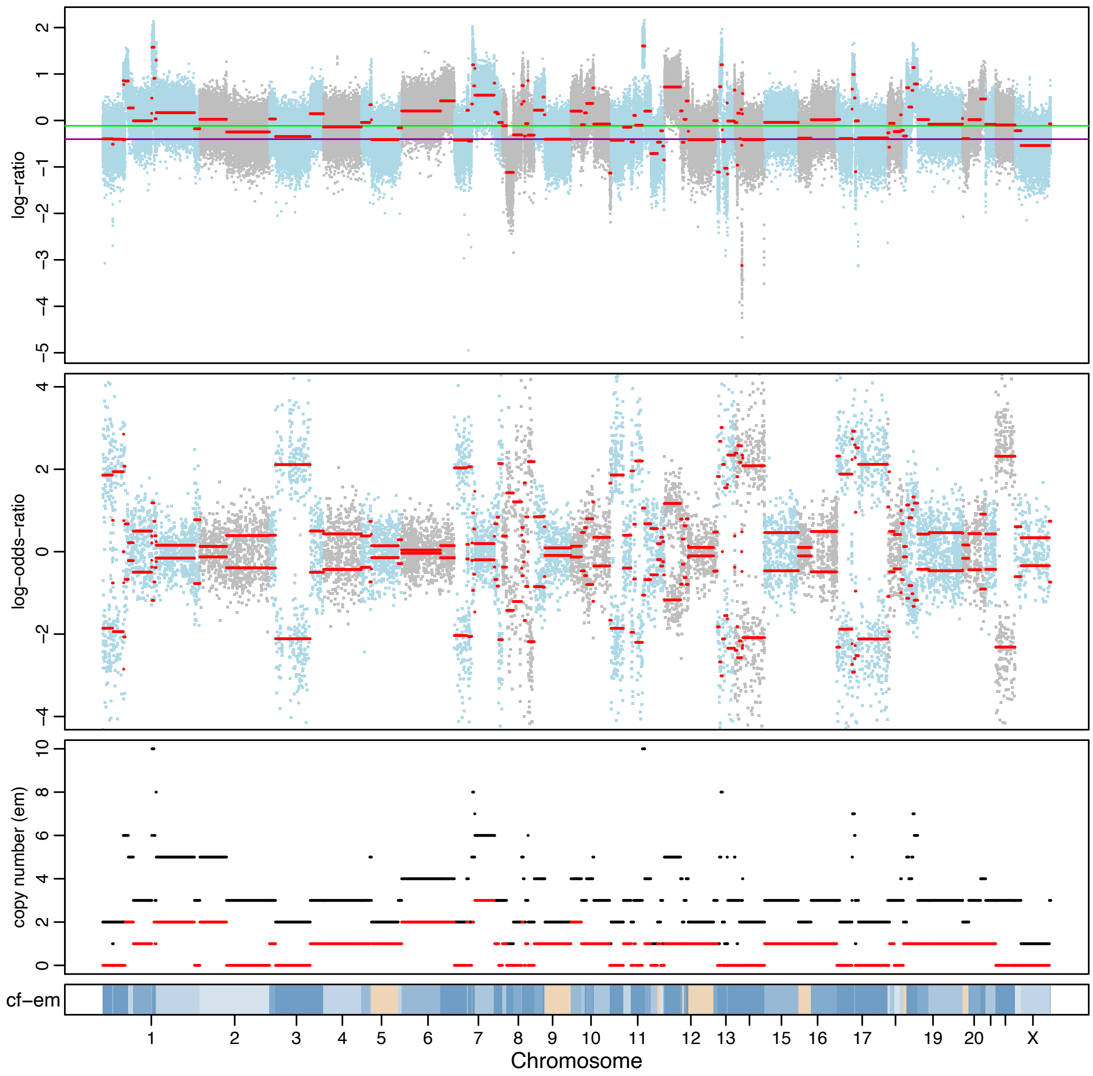

Supplementary Figure 18. Integrated visualization of FACETS analysis of WGD sample (CBCP00069). With chromosomes alternating in blue and gray, the top plot indicates copy number log-ratio (logR), and the second plot indicates allele-specific log-odds-ratio data (logOR). The third plot indicates integer (total and minor) copy number estimation (em). The bottom plot displays the cellular fraction estimation (cf-em), indicating both clonal and subclonal copy number events.

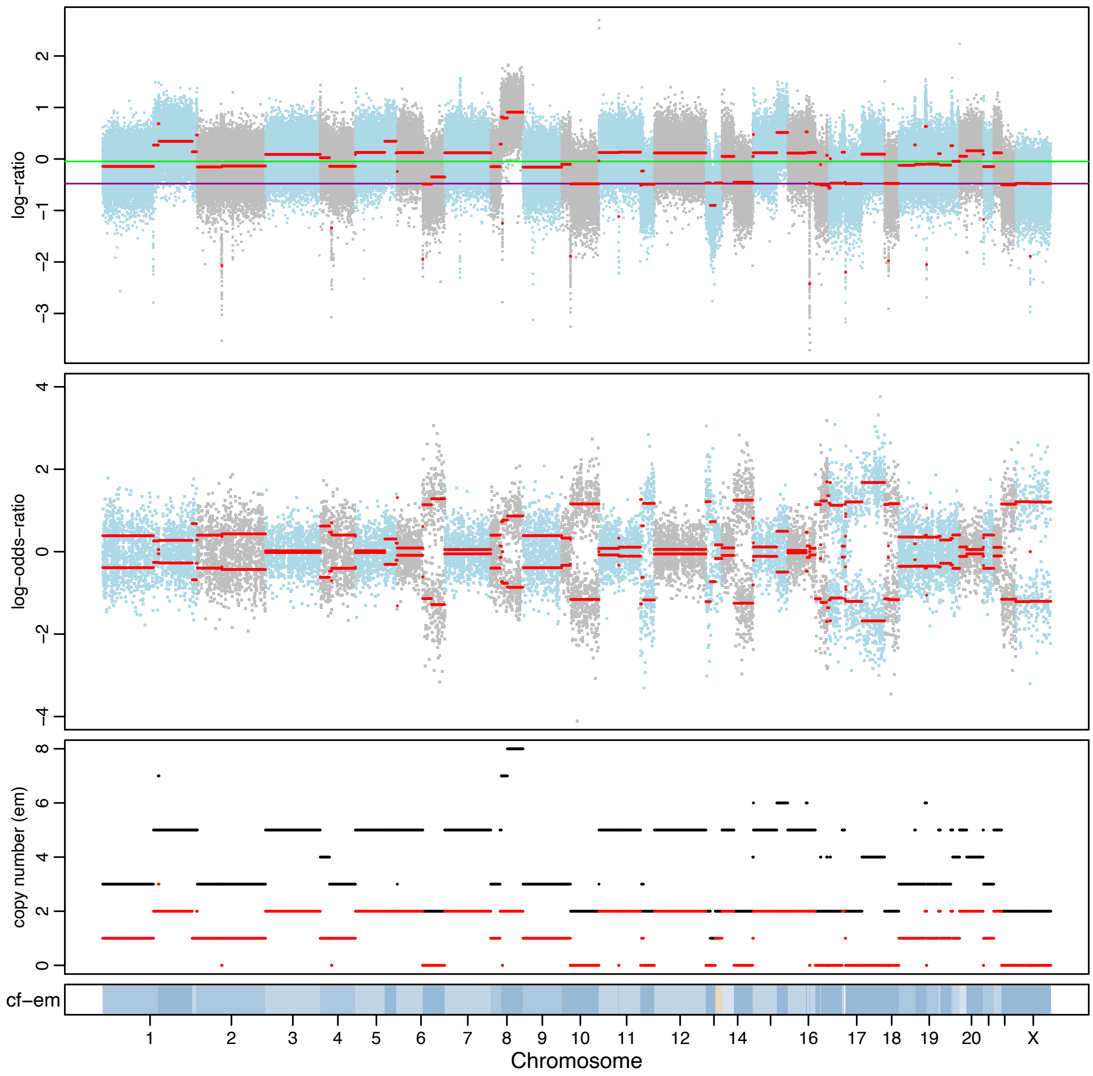

Supplementary Figure 19. Integrated visualization of FACETS analysis of WGD sample (CBCP00074). With chromosomes alternating in blue and gray, the top plot indicates copy number log-ratio (logR), and the second plot indicates allele-specific log-odds-ratio data (logOR). The third plot indicates integer (total and minor) copy number estimation (em). The bottom plot displays the cellular fraction estimation (cf-em), indicating both clonal and subclonal copy number events.

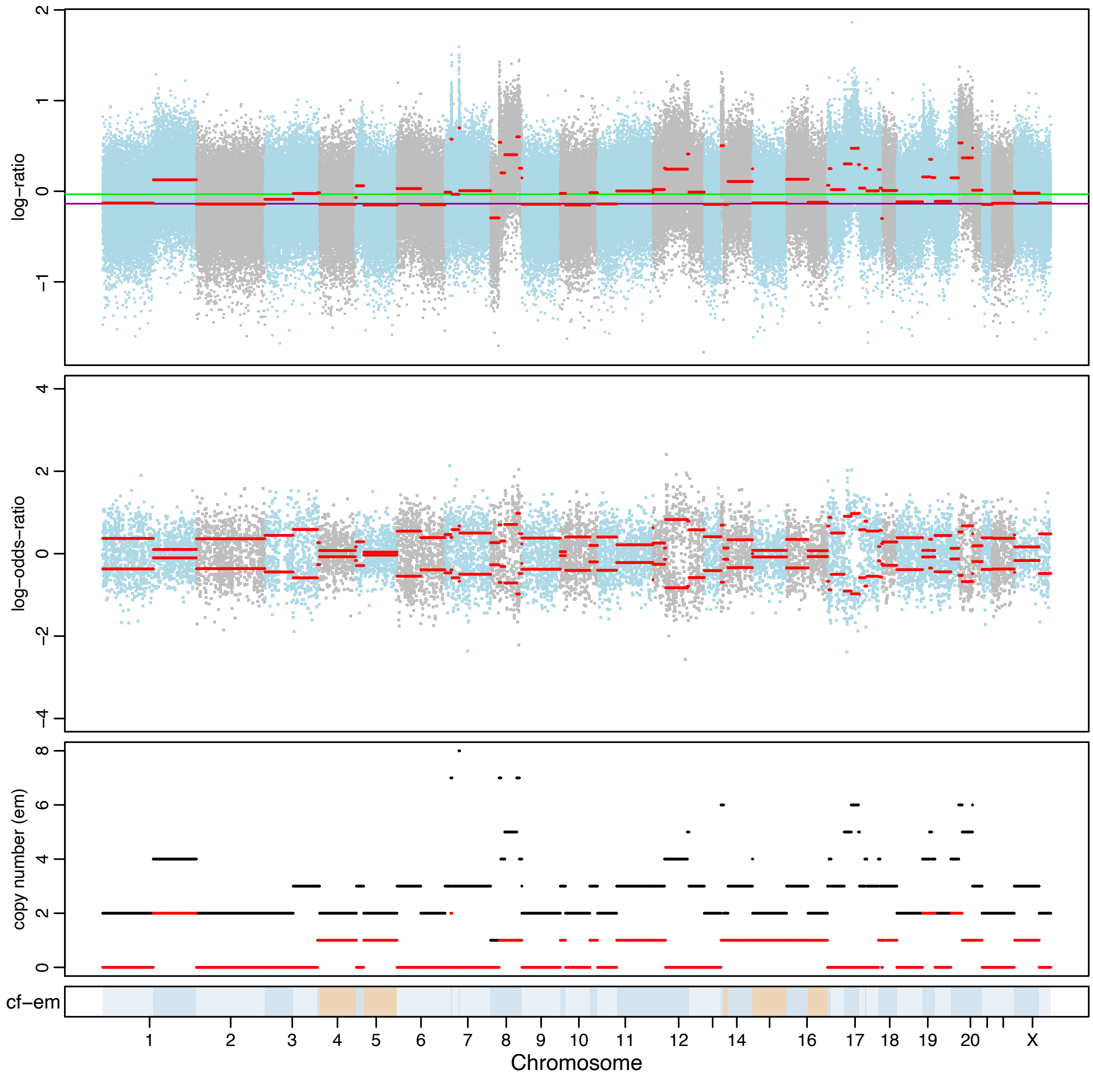

Supplementary Figure 20. Integrated visualization of FACETS analysis of WGD sample (CBCP00075). With chromosomes alternating in blue and gray, the top plot indicates copy number log-ratio (logR), and the second plot indicates allele-specific log-odds-ratio data (logOR). The third plot indicates integer (total and minor) copy number estimation (em). The bottom plot displays the cellular fraction estimation (cf-em), indicating both clonal and subclonal copy number events.

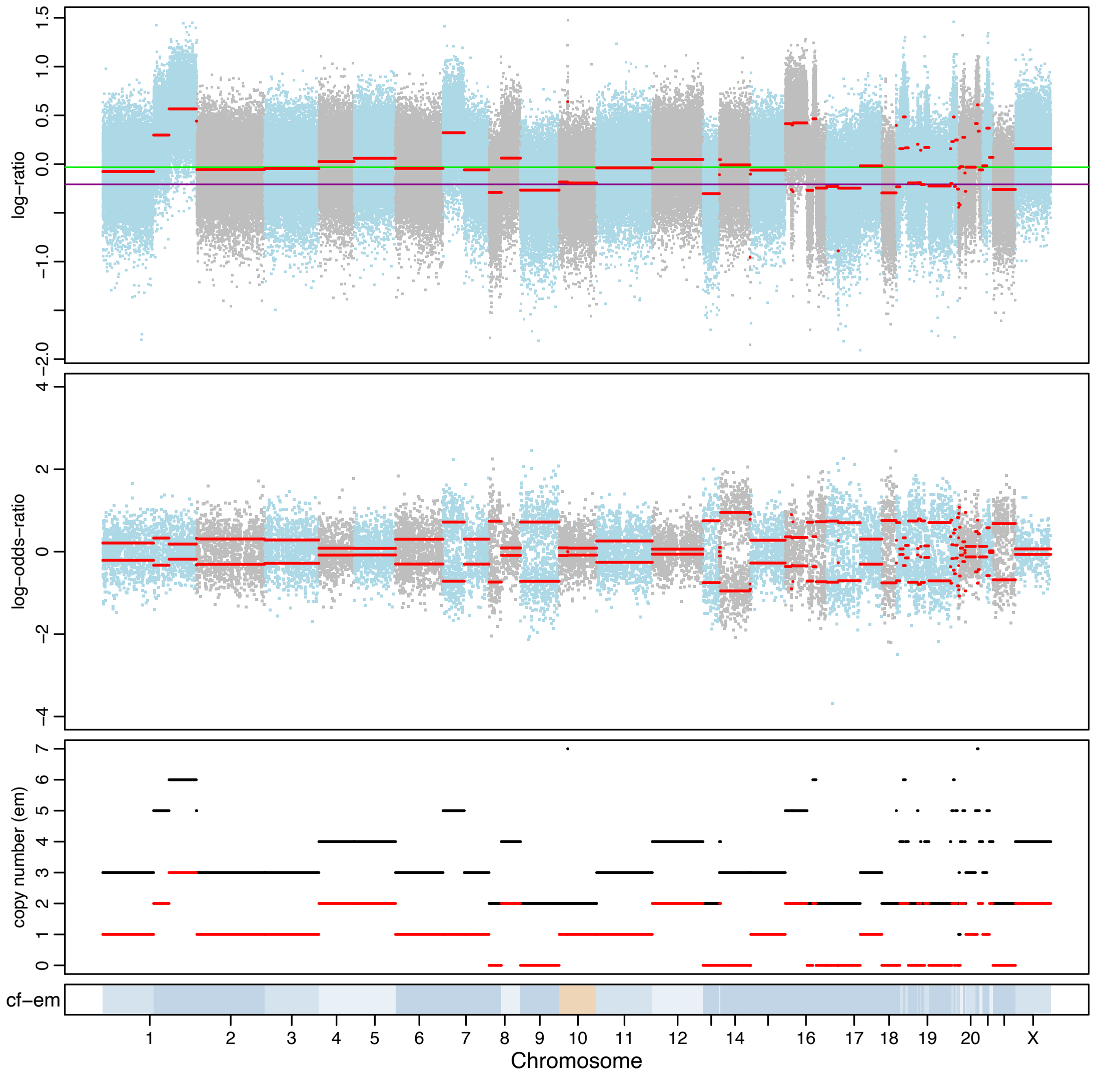

Supplementary Figure 21. Integrated visualization of FACETS analysis of WGD sample (CBCP00084). With chromosomes alternating in blue and gray, the top plot indicates copy number log-ratio (logR), and the second plot indicates allele-specific log-odds-ratio data (logOR). The third plot indicates integer (total and minor) copy number estimation (em). The bottom plot displays the cellular fraction estimation (cf-em), indicating both clonal and subclonal copy number events.

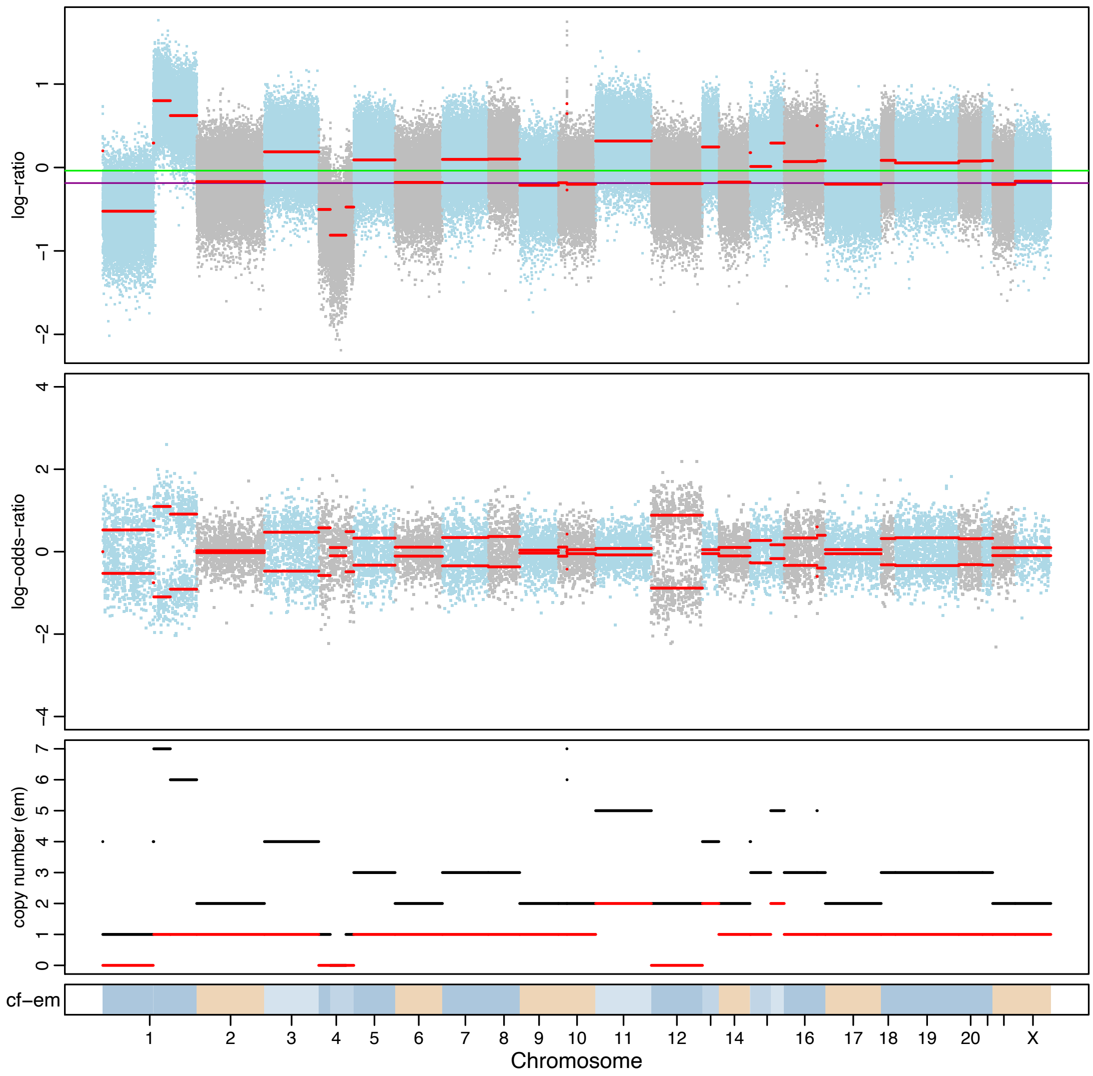

Supplementary Figure 22. Integrated visualization of FACETS analysis of WGD sample (CBCP00102). With chromosomes alternating in blue and gray, the top plot indicates copy number log-ratio (logR), and the second plot indicates allele-specific log-odds-ratio data (logOR). The third plot indicates integer (total and minor) copy number estimation (em). The bottom plot displays the cellular fraction estimation (cf-em), indicating both clonal and subclonal copy number events.

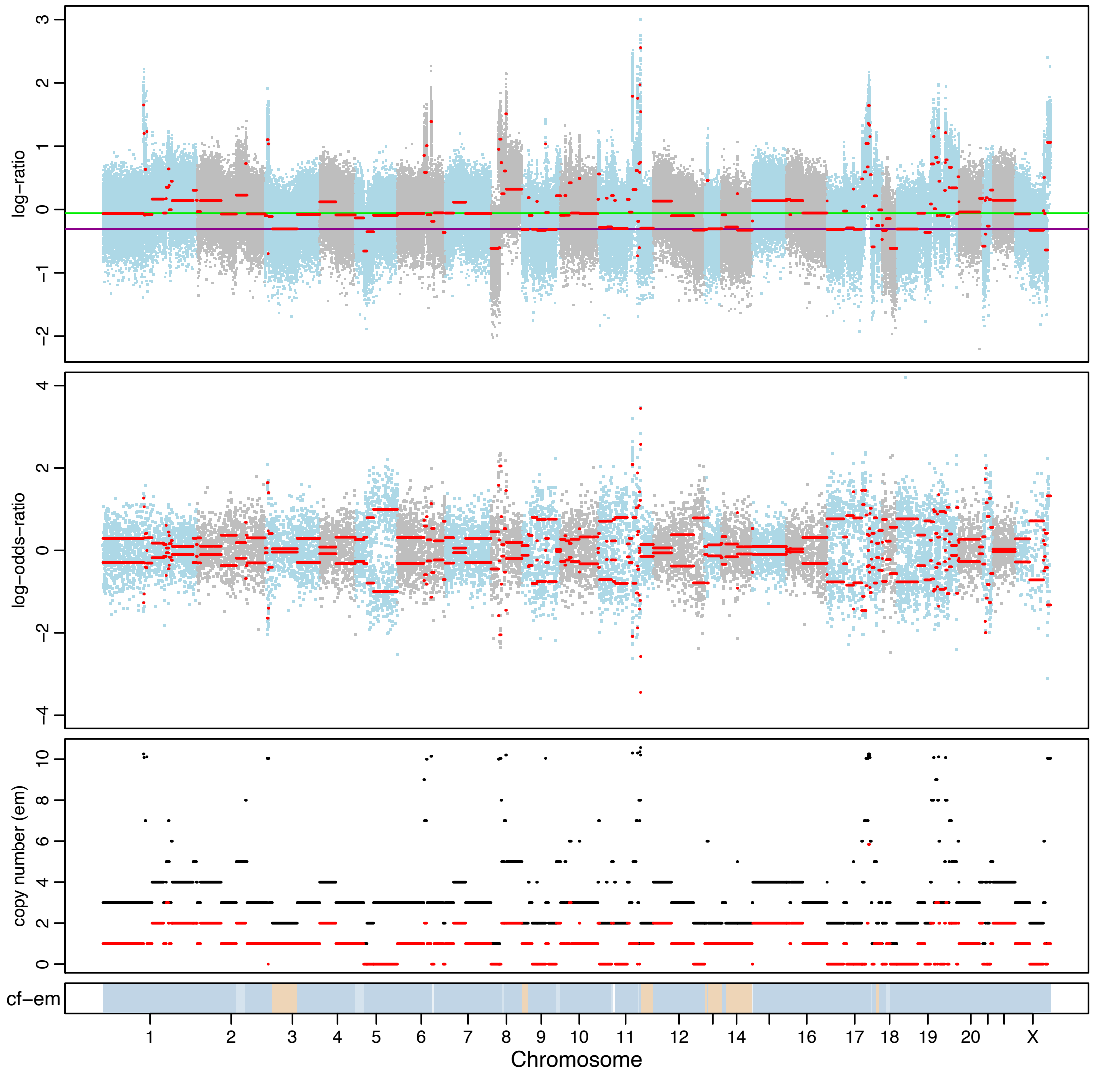

Supplementary Figure 23. Integrated visualization of FACETS analysis of WGD sample (CBCP00257). With chromosomes alternating in blue and gray, the top plot indicates copy number log-ratio (logR), and the second plot indicates allele-specific log-odds-ratio data (logOR). The third plot indicates integer (total and minor) copy number estimation (em). The bottom plot displays the cellular fraction estimation (cf-em), indicating both clonal and subclonal copy number events.

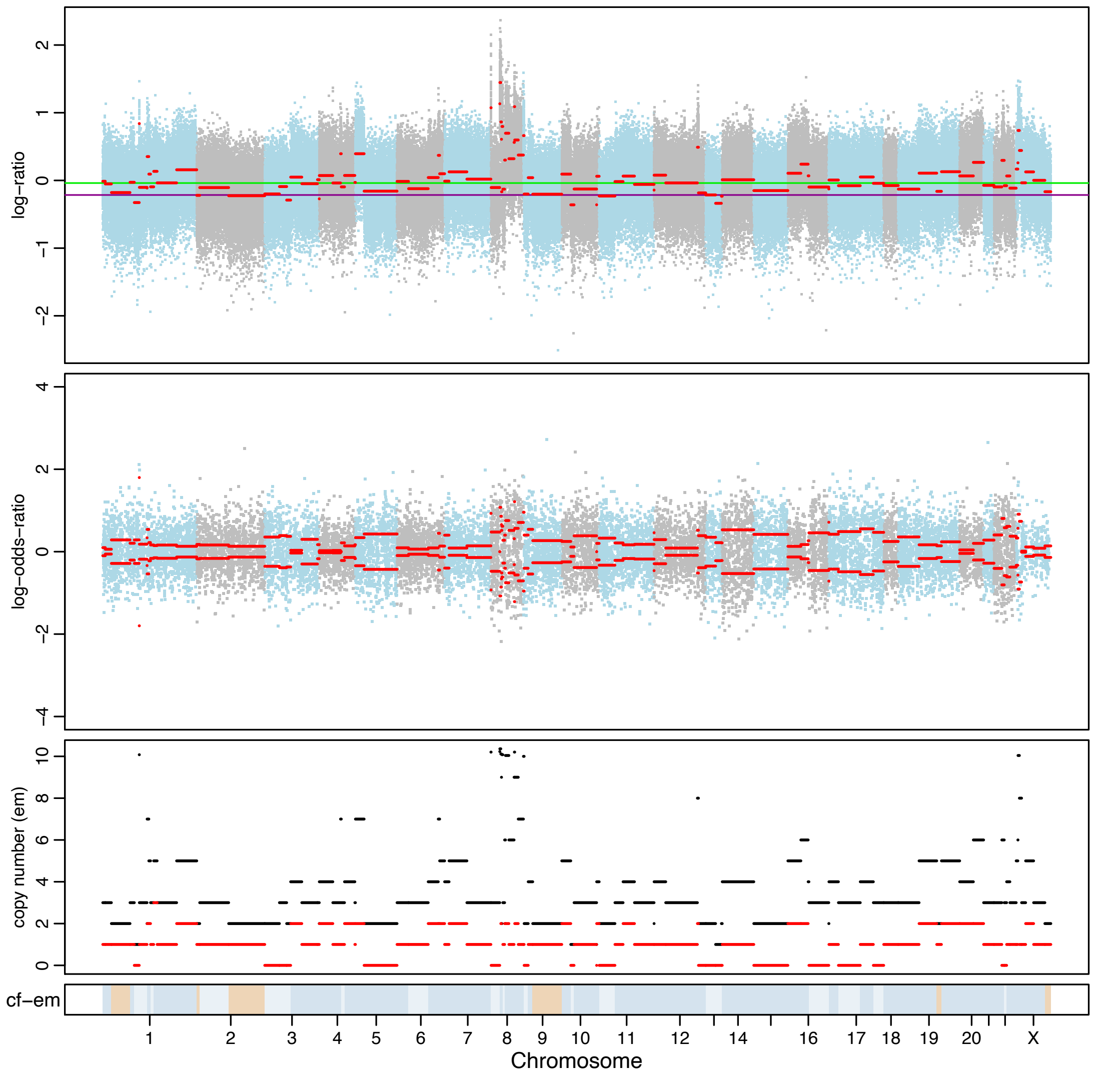

Supplementary Figure 24. Integrated visualization of FACETS analysis of WGD sample (CBCP00262). With chromosomes alternating in blue and gray, the top plot indicates copy number log-ratio (logR), and the second plot indicates allele-specific log-odds-ratio data (logOR). The third plot indicates integer (total and minor) copy number estimation (em). The bottom plot displays the cellular fraction estimation (cf-em), indicating both clonal and subclonal copy number events.

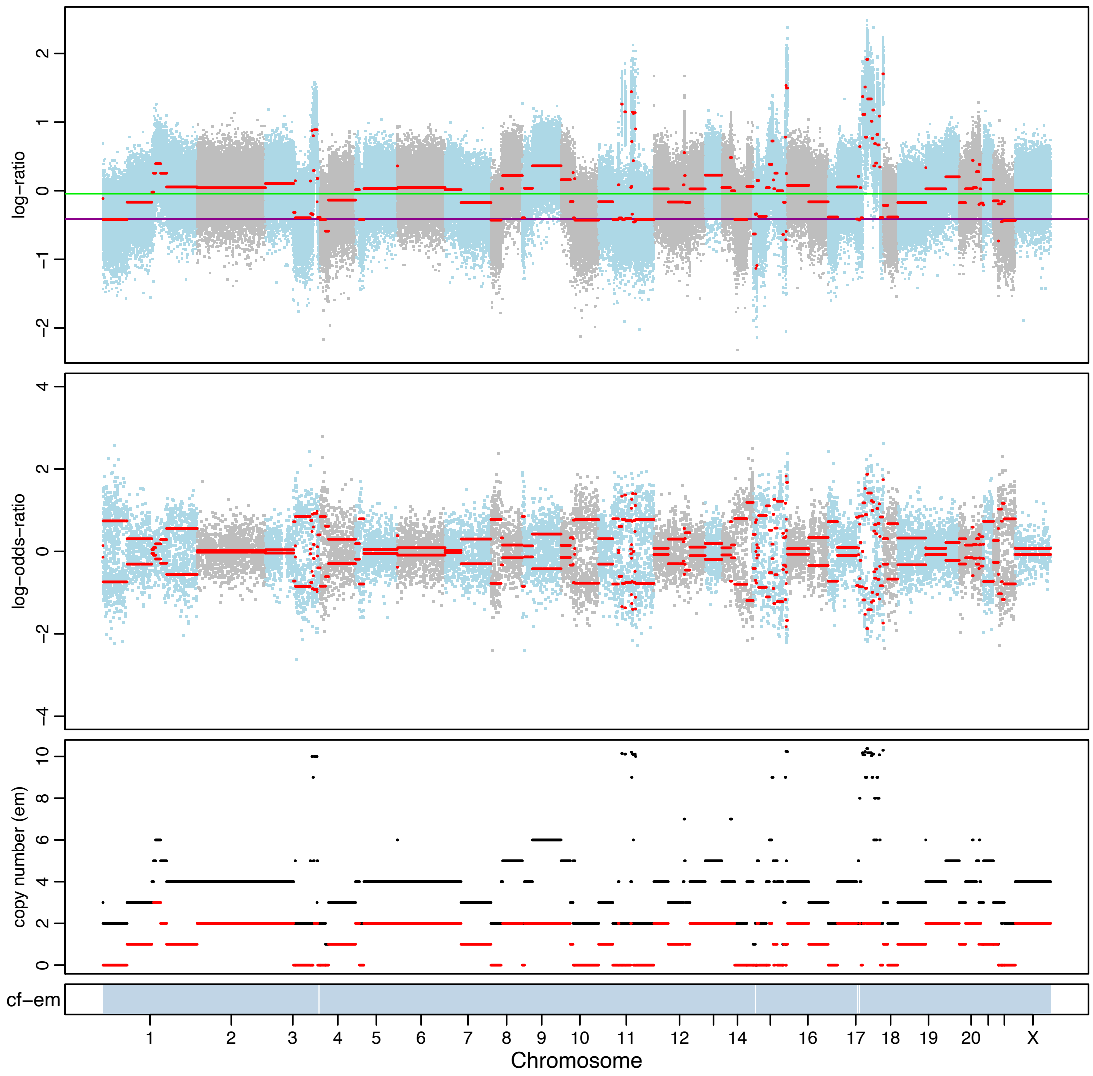

Supplementary Figure 25. Integrated visualization of FACETS analysis of WGD sample (CBCP00268). With chromosomes alternating in blue and gray, the top plot indicates copy number log-ratio (logR), and the second plot indicates allele-specific log-odds-ratio data (logOR). The third plot indicates integer (total and minor) copy number estimation (em). The bottom plot displays the cellular fraction estimation (cf-em), indicating both clonal and subclonal copy number events.

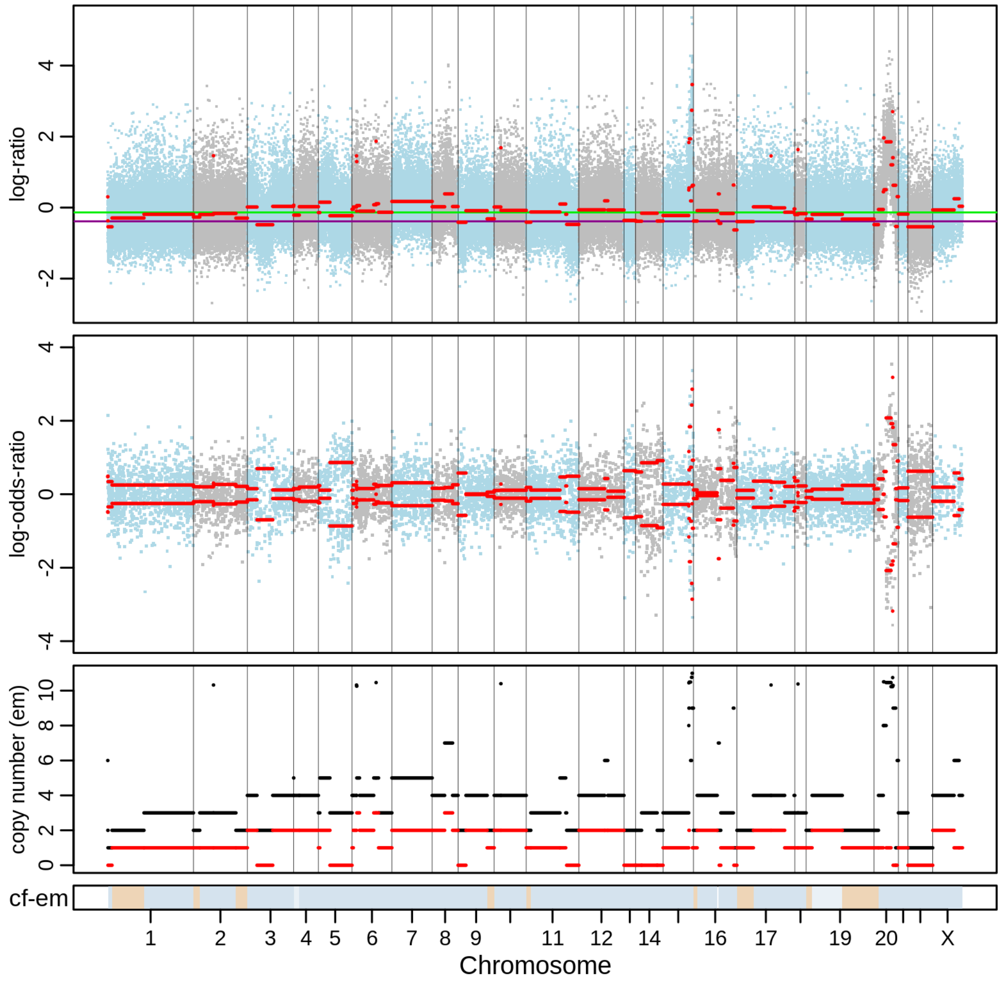

Supplementary Figure 26. Integrated visualization of FACETS analysis of WGD sample (CBCP00271). With chromosomes alternating in blue and gray, the top plot indicates copy number log-ratio (logR), and the second plot indicates allele-specific log-odds-ratio data (logOR). The third plot indicates integer (total and minor) copy number estimation (em). The bottom plot displays the cellular fraction estimation (cf-em), indicating both clonal and subclonal copy number events.

Supplementary Note 1

Discussion of BCTW-specific SMGs

An additional contribution from this work was the identification of 13 well-known cancer genes in the BCTW population, as well as 3 novel SMGs: *COMP*, *ERN1*, and *PIGT*. *COMP* contributes to the severity of the disease by metabolic switching and increasing invasiveness to reduce survival in breast cancer patients[1]. Inactivating *ERN1* can enhance the effectiveness of current chemotherapeutics in therapeutic strategies for TNBC[2]. Notably, *PIGT* function in breast carcinogenesis has not been well established. However, these genes should be further validated in more BCTW samples in the future. In addition to these novel genes, *SF3B1* was found to have elevated mutation rates in BCTW; previous reports showed that mutations in *SF3B1* were mostly acquired around the highly conserved HEAT repeats and that this gene is considered a potential therapeutic target in breast cancer[3]. Our study provides a rigorous framework in which to elucidate the specific mutational profiles for BCTW patients and offers a unique perspective and some unique advantages for studying cancer genomics and cancer heterogeneity.

Supplementary References

1. Englund, E. *et al.* Cartilage oligomeric matrix protein contributes to the development and metastasis of breast cancer. *Oncogene* **35**, 5585-5596 (2016).
2. Logue, S.E. *et al.* Inhibition of IRE1 RNase activity modulates the tumor cell secretome and enhances response to chemotherapy. *Nat Commun* **9**, 3267 (2018).
3. Maguire, S.L. *et al.* SF3B1 mutations constitute a novel therapeutic target in breast cancer. *J Pathol* **235**, 571-80 (2015).
