# Supplementary material for: Differential whole-genome doubling and homologous recombination deficiencies across breast cancer subtypes from the Taiwanese population
Source: Commun Biol. 2021 Sep 9;4:1052. doi: 10.1038/s42003-021-02597-x (PMC8429690; doi:10.1038/s42003-021-02597-x)
Supplement: Supplementary file 3 — Description of Supplementary Files [file 42003_2021_2597_MOESM3_ESM.pdf]

Description of Additional Supplementary Files

Supplementary Data 1: The number of indels called in each sample

Supplementary Data 2: Sequencing and sample quality metric for whole-genome doubling samples

Supplementary Data 3: The bootstrapping data for 95% confidence interval of mutational frequencies in each population
