# Supplementary material for: Differential whole-genome doubling and homologous recombination deficiencies across breast cancer subtypes from the Taiwanese population
Source: Commun Biol. 2021 Sep 9;4:1052. doi: 10.1038/s42003-021-02597-x (PMC8429690; doi:10.1038/s42003-021-02597-x)
Supplement: Supplementary file 7 — Reporting Summary [file 42003_2021_2597_MOESM7_ESM.pdf]

# nature portfolio

Corresponding author(s): Eric Y. Chuang

Last updated by author(s): Jul 21, 2021

# Reporting Summary

Nature Portfolio wishes to improve the reproducibility of the work that we publish. This form provides structure for consistency and transparency in reporting. For further information on Nature Portfolio policies, see our Editorial Policies and the Editorial Policy Checklist.

## Statistics

For all statistical analyses, confirm that the following items are present in the figure legend, table legend, main text, or Methods section.

| n/a | Confirmed | |
|---|---|---|
| ☐ | ☒ | The exact sample size (*n*) for each experimental group/condition, given as a discrete number and unit of measurement |
| ☐ | ☒ | A statement on whether measurements were taken from distinct samples or whether the same sample was measured repeatedly |
| ☐ | ☒ | The statistical test(s) used AND whether they are one- or two-sided<br>*Only common tests should be described solely by name; describe more complex techniques in the Methods section.* |
| ☐ | ☒ | A description of all covariates tested |
| ☒ | ☐ | A description of any assumptions or corrections, such as tests of normality and adjustment for multiple comparisons |
| ☐ | ☒ | A full description of the statistical parameters including central tendency (e.g. means) or other basic estimates (e.g. regression coefficient) AND variation (e.g. standard deviation) or associated estimates of uncertainty (e.g. confidence intervals) |
| ☐ | ☒ | For null hypothesis testing, the test statistic (e.g. *F*, *t*, *r*) with confidence intervals, effect sizes, degrees of freedom and *P* value noted<br>*Give P values as exact values whenever suitable.* |
| ☒ | ☐ | For Bayesian analysis, information on the choice of priors and Markov chain Monte Carlo settings |
| ☒ | ☐ | For hierarchical and complex designs, identification of the appropriate level for tests and full reporting of outcomes |
| ☒ | ☐ | Estimates of effect sizes (e.g. Cohen's *d*, Pearson's *r*), indicating how they were calculated |

*Our web collection on statistics for biologists contains articles on many of the points above.*

## Software and code

Policy information about availability of computer code

| | |
|---|---|
| Data collection | An Illumina Hiseq4000 instrument was used to obtain raw whole-exome sequencing data (fastq files). |
| Data analysis | Alignment: BWA-MEM (v.0.7.15)<br>BAM sorting and duplicates marking: Picard module (v.2.6.0)<br>Indel realignment and base quality score recalibration: GATK (v.3.7)<br>Somatic SNV and indel calling: GATK MuTect2<br>Post processing: GATK, D-ToxoG, DKFZ Bias Filter, DeTiN, ABSOLUTE, and SigProfilerMatrixGenerator<br>Variant annotation: Variant Effect Predictor (v.89) and vcf2maf script<br>Significantly mutated gene: dNdScv, OncodriveCLUST, OncodriveFML, and 20/20+<br>Copy number variation: FACETS (v.0.5.2) and GISTIC2 (v.2.0.23)<br>All of these tools are publicly available.<br>Statistical and other analyses were performed using software R (version 3.5.1, included packages: MutationalPatterns, NMF, and scarHRD) and described in the material and method section. |

For manuscripts utilizing custom algorithms or software that are central to the research but not yet described in published literature, software must be made available to editors and reviewers. We strongly encourage code deposition in a community repository (e.g. GitHub). See the Nature Portfolio guidelines for submitting code & software for further information.

## Data

Policy information about availability of data

All manuscripts must include a data availability statement. This statement should provide the following information, where applicable:

- Accession codes, unique identifiers, or web links for publicly available datasets
- A description of any restrictions on data availability
- For clinical datasets or third party data, please ensure that the statement adheres to our policy

Raw whole-exome sequencing data used in this study can be found in the NCBI database under the BioProject accession PRJNA729775. The molecular and clinical data of breast cancer patients from the TCGA cohort are available in the following repositories: TCGA BRCA: https://portal.gdc.cancer.gov/ (MuTect2 MAF file) and cBioPortal: https://www.cbioportal.org/ (clinical data).

# Field-specific reporting

Please select the one below that is the best fit for your research. If you are not sure, read the appropriate sections before making your selection.

☒ Life sciences ☐ Behavioural & social sciences ☐ Ecological, evolutionary & environmental sciences

For a reference copy of the document with all sections, see nature.com/documents/nr-reporting-summary-flat.pdf

# Life sciences study design

All studies must disclose on these points even when the disclosure is negative.

| | |
|---|---|
| Sample size | No sample size estimation was performed. We analyzed whole-exome sequencing data from 116 breast cancer patients in the Taiwanese population and there were no more suitable data of Taiwanese cohort in existence that the authors knew of to conduct these analyses. TCGA breast cancer data was also included in this study. |
| Data exclusions | TCGA patients without mutation or race information were excluded from the downstream comparisons (Fig. 1) via post-filtering strategies based on the TCGA MC3 project. |
| Replication | We did not perform any analysis that required replication. |
| Randomization | We did not perform any analysis that required randomization. |
| Blinding | We did not perform any analysis that required blinding method. |

# Reporting for specific materials, systems and methods

We require information from authors about some types of materials, experimental systems and methods used in many studies. Here, indicate whether each material, system or method listed is relevant to your study. If you are not sure if a list item applies to your research, read the appropriate section before selecting a response.

## Materials & experimental systems

| n/a | Involved in the study | |
|---|---|---|
| ☒ | ☐ | Antibodies |
| ☒ | ☐ | Eukaryotic cell lines |
| ☒ | ☐ | Palaeontology and archaeology |
| ☒ | ☐ | Animals and other organisms |
| ☐ | ☒ | Human research participants |
| ☒ | ☐ | Clinical data |
| ☒ | ☐ | Dual use research of concern |

## Methods

| n/a | Involved in the study | |
|---|---|---|
| ☒ | ☐ | ChIP-seq |
| ☒ | ☐ | Flow cytometry |
| ☒ | ☐ | MRI-based neuroimaging |

## Human research participants

Policy information about studies involving human research participants

| | |
|---|---|
| Population characteristics | Patients clinically diagnosed breast cancer and underwent surgical resection at 1) Lotung Poh-Ai Hospital, Yilan County, Taiwan; 2) Cathay General Hospital, Taipei, Taiwan; 3) Kaohsiung Medical University Hospital, Kaohsiung, Taiwan; and 4) Cheng-Ching Hospital, Taichung, Taiwan. |
| Recruitment | Patients were identified based on inclusion criteria listed above. |
| Ethics oversight | All samples were collected with informed consent under IRB-approved protocols of these hospitals. |

Note that full information on the approval of the study protocol must also be provided in the manuscript.
